# The biology of habitat dominance; can microbes behave as weeds?

**DOI:** 10.1111/1751-7915.12027

**Published:** 2013-01-22

**Authors:** Jonathan A Cray, Andrew N W Bell, Prashanth Bhaganna, Allen Y Mswaka, David J Timson, John E Hallsworth

**Affiliations:** School of Biological Sciences, MBC, Queen's University BelfastBelfast, BT9 7BL, Northern Ireland, UK

## Abstract

**Summary:**

Competition between microbial species is a product of, yet can lead to a reduction in, the microbial diversity of specific habitats. Microbial habitats can resemble ecological battlefields where microbial cells struggle to dominate and/or annihilate each other and we explore the hypothesis that (like plant weeds) some microbes are genetically hard-wired to behave in a vigorous and ecologically aggressive manner. These ‘microbial weeds’ are able to dominate the communities that develop in fertile but uncolonized – or at least partially vacant – habitats via traits enabling them to out-grow competitors; robust tolerances to habitat-relevant stress parameters and highly efficient energy-generation systems; avoidance of or resistance to viral infection, predation and grazers; potent antimicrobial systems; and exceptional abilities to sequester and store resources. In addition, those associated with nutritionally complex habitats are extraordinarily versatile in their utilization of diverse substrates. Weed species typically deploy multiple types of antimicrobial including toxins; volatile organic compounds that act as either hydrophobic or highly chaotropic stressors; biosurfactants; organic acids; and moderately chaotropic solutes that are produced in bulk quantities (e.g. acetone, ethanol). Whereas ability to dominate communities is habitat-specific we suggest that some microbial species are archetypal weeds including generalists such as: *Pichia anomala*, *Acinetobacter* spp. and *Pseudomonas putida*; specialists such as *Dunaliella salina*, *Saccharomyces cerevisiae*, *Lactobacillus* spp. and other lactic acid bacteria; freshwater autotrophs *Gonyostomum semen* and *Microcystis aeruginosa*; obligate anaerobes such as *Clostridium acetobutylicum*; facultative pathogens such as *Rhodotorula mucilaginosa*, *Pantoea ananatis* and *Pseudomonas aeruginosa*; and other extremotolerant and extremophilic microbes such as *Aspergillus* spp., *Salinibacter ruber* and *Haloquadratum walsbyi*. Some microbes, such as *Escherichia coli*, *Mycobacterium smegmatis* and *Pseudoxylaria* spp., exhibit characteristics of both weed and non-weed species. We propose that the concept of nonweeds represents a ‘dustbin’ group that includes species such as *Synodropsis* spp., *Polypaecilum pisce*, *Metschnikowia orientalis*, *Salmonella* spp., and *Caulobacter crescentus*. We show that microbial weeds are conceptually distinct from plant weeds, microbial copiotrophs, *r*-strategists, and other ecophysiological groups of microorganism. Microbial weed species are unlikely to emerge from stationary-phase or other types of closed communities; it is open habitats that select for weed phenotypes. Specific characteristics that are common to diverse types of open habitat are identified, and implications of weed biology and open-habitat ecology are discussed in the context of further studies needed in the fields of environmental and applied microbiology.

## Introduction

As the collective metabolism and ecological activities of microorganisms determine the health and sustainability of life on Earth it is essential to understand the function of both individual microbes and in their communities. The cellular systems of an insignificant portion of microbial species have been intensively characterized at the levels of biochemistry, cell biology, genomics and systems biology; and a substantial body of (top-down) studies has been published in the field of microbial ecology in relation to species interactions, community succession and environmental metagenomics. There are, however, some fundamental questions that remain unanswered in relation to the behaviour of microbial species within communities: what type of biology, for instance, enables microbes to dominate entire communities and their habitats and thereby determine levels of biodiversity in specific environments?

Plant species that primarily inhabit freshly disturbed habitats – known as weeds – are characterized by vigorous growth; tolerance to multiple stresses; exceptional reproduction, dispersal and survival mechanisms; a lack of specific environmental requirements; production of phytotoxic chemicals; and/or other competitive strategies (Table 1). The biology of plant weeds, including their phenotypic and genetic traits, has been the subject of extensive study over the past 100 years and is now relatively well characterized (Table 1; Long, 1932; Anderson, 1952; Salisbury, 1961; Hill, 1977; Mack *et al*., 2000; Schnitzler and Bailey, 2008; Bakker *et al*., 2009; Chou, 2010; Wu *et al*., 2011; Liberman *et al*., 2012). We hypothesize that weed biology is also pertinent to microbial species, but that the defining characteristic of microbial weeds is their ability to *dominate* their respective communities. In the field of plant ecology it is open habitats (such as freshly exposed fertile soil) that facilitate the emergence of weed species both within specific ecosystems and across evolutionary timescales. We propose that weed behaviour is equally prevalent in some microbial habitats, that open microbial habitats promote the emergence of microbial weed species, and that microbial weed biology represents a potent ecological and evolutionary mechanism of change for some microbial species and their communities.

**Figure 1:**
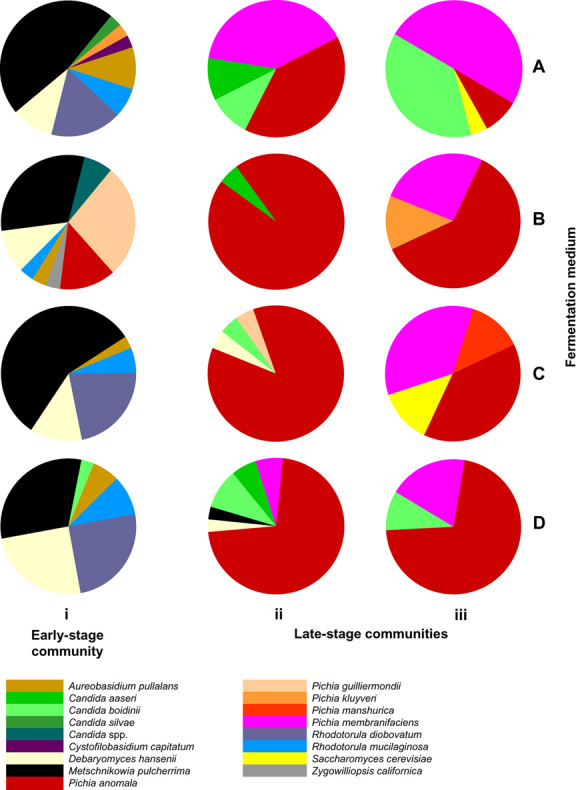
Percentage species composition during olive fermentations at (i) 2 days, (ii) 17 days and (iii) 35 days over a range of conditions: (A) with added NaCl (6% w/v); (B) added NaCl and glucose (6% and 0.5% w/v respectively); (C) added NaCl and lactic acid (6% w/v and 0.2% v/v respectively); and (D) NaCl, glucose and lactic acid (6% and 0.5% w/v, and 0.2% v/v respectively). Data (from Nisiotou *et al*., 2010) were obtained by DGGE.

**Figure 2:**
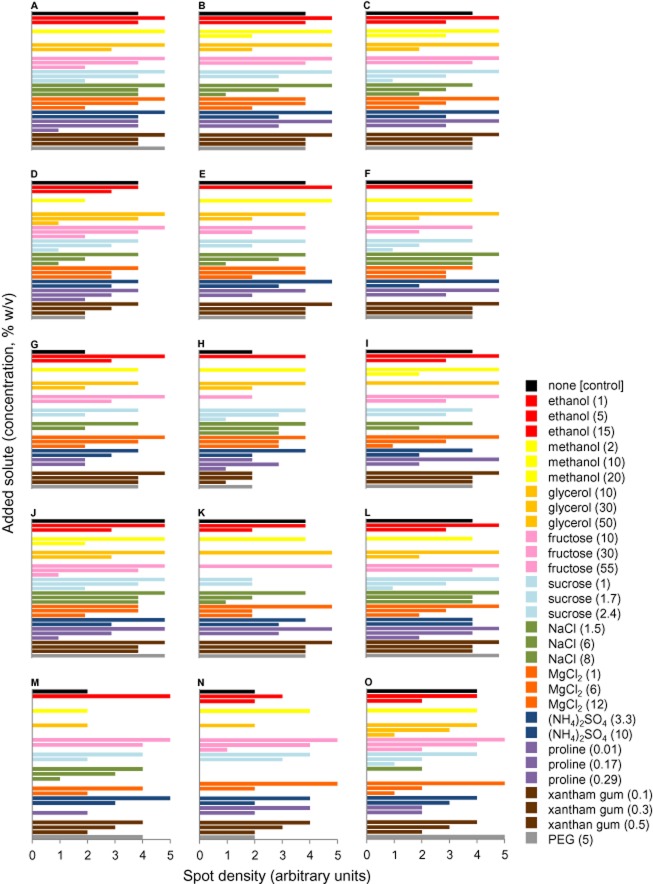
Growth of *Saccharomyces cerevisiae* strains: (A) CCY-21-4-13, (B) Alcotec 8 and (C) CBS 6412 and other yeast species: (D) *Candida etchellsii* (UWOPS 01–168.3), (E) *Cryptococcus terreus* (PB4), (F) *Debaryomyces hansenii* (UWOPS 05-230.3), (G) *Hansenula* (*Ogataea*) *polymorpha* (CBS 4732), (H) *Hortaea werneckii* (MZKI B736), (I) *Kluyveromyces marxianus* (CBS 712), (J) *Pichia (Kodamaea) ohmeri* (UWOPS 05-228.2), (K) *Pichia* (*Komagataella*) *pastoris* (CBS 704), (L) *Pichia sydowiorum* (UWOPS 03-414.2), (M) *Rhodotorula creatinivora* (PB7), (N) *Saccharomycodes ludwigii* (UWOPS 92–218.4) and (O) *Zygosaccharomyces rouxii* (CBS 732) on a range of media at 30°C. These were: malt-extract, yeast-extract phosphate agar (MYPiA) without added solutes (control), and MYPiA supplemented with diverse stressors – ethanol, methanol, glycerol, fructose, sucrose, NaCl, MgCl_2_, ammonium sulfate, proline, xantham gum and polyethylene glycol (PEG) 8000 – over a range of concentrations as shown in key (values indicate %, w/v). A standard Spot Test was carried out (see Chin *et al*., 2010; Toh *et al*., 2001) that was modified from Albertyn and colleagues (1994) for stress phenotype characterization (see Table 5). Colony density was assessed after an incubation time of 24 h on a scale of 0–5 arbitrary units (Chin *et al*., 2010). Cultures were obtained from (for strain A) the Culture Collection of Yeasts (CCY, Slovakia); (strain B) Hambleton Bard Ltd, Chesterfield, UK; (strains C, G, I, K, O) the Centraalbureau voor Schimmelcultures (CBS, the Netherlands); (strains D, F, J, L, N) the University of Western Ontario Plant Sciences Culture Collection (UWOPS, Canada); (strains E, M) were obtained from Dr Rosa Margesin, Institute of Microbiology, Leopold Franzens University, Austria; and (strain H) the Microbial Culture Collection of National Institute of Chemistry (MZKI, Slovenia). All Petri plates containing the same medium were sealed in a polythene bag to maintain water; all experiments were carried out in duplicate, and plotted values are the means of independent treatments.

**Table 1:** A comparison of plant weeds and microbial weeds.^a^

	Plant weeds	Microbial weeds	References (plants/microbes)
*Definition*	Plant species that primarily grow in open (plant) habitats^b^	Microbial species able to dominate communities that develop in open (microbial) habitats^c^	Baker, 1965; Hill, 1977/current article; Cordero *et al*., 2012
*Ability to rapidly occupy available space*	Important trait	Essential trait	Hill, 1977/current article
*Overall vigour*^d^ *and competitive ability*	Typically vigorous; competitive ability usually superior to that of crop plants	Exceptional vigour and competitive ability	Hill, 1977/Figs 1 and 2; Tables S1 and 2–5
*Primary competitors*	Intra-specific competition can be stronger than inter-specific competition	Intra-and inter-specific competition are likely to be stronger if dominance results from efficient resource acquisition or antimicrobial substances respectively	Hill, 1977/current article
*Resource acquisition and storage*	Acquisition and/or storage of water, light, nutrients etc. typically excellent	Acquisition and/or storage of nutrients, water and/or light typically excellent	Hill, 1977; Oatham, 1997; Sadeghi *et al*., 2007/Table5
*Reproduction and dispersal*	Exceptional^e^	May not differ from non-weed species	Hill, 1977/current article
*Obligatory associations or interactions with other organisms*	Uncommon/atypical	Uncommon/atypical	Hill, 1977/current article
*Ubiquity*	Many species are environmentally ubiquitous	Not necessarily widespread outside the habitat which they dominate	Hill, 1977/current article
*Germination*	No special requirements; high tolerance to diverse stresses (see below)	No special requirements; high tolerance to diverse stresses (see below)	Hill, 1977/current article
*Longevity of dormant propagules*	Exceptional	May not differ from non-weeds	Hill, 1977/current article
*Able to regenerate from fragments*	Yes, especially root fragments	Not relevant	Hill, 1977/current article
*Maintenance of growth rate during nutrient limitation*	Some species, e.g. black-grass (*Alopecurus mysuroides*) on low-potassium soils	Many (photosynthetic as well as some heterotrophic) species	Hill, 1977/Table5; Benning, 1998
*Capable of creating a zone-of-inhibition*	Not known	Yes, in some instances	[Not known]/Fleming, 1929
*Secretion of toxic substances*	Some species produce phytotoxic inhibitors (exuded from leaves, roots, and/or necromass)	All species produce antimicrobial toxins (secreted into the extracellular environment)^f^	Chou, 2010/Table6
*Production of volatile organic compounds (VOCs) that act as stressors*	Some hydrophobic and highly chaotropic compounds act as phytotoxic stressors (e.g. β-caryophellene)	Many species produce a spectrum of VOCs that can inhibit metabolic activity at concentrations in the nanomolar or low millimolar range^f^	Wang *et al*., 2010; Inderjit *et al*., 2011; Tsubo *et al*., 2012/Table6

*Secretion of biosurfactants that act as stressors*	Not known	Some species produce biosurfactants; substances that can solubilize hydrophobic substrates and act as cellular stressors^f^	[Not known]/Tables3 and e; Nitschke *et al*., 2011
*Bulk production, and secretion, of moderately chaotropic substances*	Not known	Some species produce ethanol, butanol, acetone etc. that are potent cellular stressors at millimolar and molar concentrations^f^	[Not known]/Table6
*Saprotrophic activity resulting in toxic/stressful breakdown products*	Not known	Some species degrade macromolecules to release inhibitory catabolic products (e.g. chaotropic stressors such as phenol, catechol and vanillin^g^)	[Not known]/Park *et al*., 2004; 2007; Zuroff and Curtis, 2012
*Acidification of habitat*	Not known	Some species, via bulk production of organic acids^f^	[Not known]/Table6
*Secretion of aggressive enzymes*	Unlikely	Some species^f^	[Not known]/Table6; Walker, 2011
*Tolerance to environmental stresses*^h^	Tolerant to multiple, habitat-relevant stress parameters	Tolerant to multiple, habitat-relevant stress parameters	Hill, 1977/Fig. 2, Tables2–4 and 6
*Resistance to toxins and/or stressors of biotic origin*^h^	Likely	Yes, most species	Hill, 1977/Fig. 2; Tables2–4 and 6
*Phenotypic plasticity and intra-specific variation*	Greater than that of crop plants	Likely to be above average (e.g. in relation to stress biology)	Hill, 1977/current article; see references in Todd *et al*., 2004
*Resistance to viruses, predators, and/or grazers*	Some species can tolerate, resist, and/or repel viruses, sucking insects and grazing animals; and may contain poisonous substances – or have morphological adaptations such as spines – that minimize losses through grazing	Some strains/species can tolerate, resist, repel and/or avoid viruses, grazing plankton and arthropods etc; may contain toxic metabolites or have behavioural adaptations – e.g. formation of large microcolonies – that minimize losses through grazing/predation; and may inhabit environments that are too extreme for predatory species	Thurston *et al*., 2001; Ralphs, 2002; Shukurov *et al*., 2012/Table6
*Karyotype*	Commonly of hybrid origin, polyploid or aneuploid	Frequency of hybrid origin, polyploidy and aneuploidy not yet tested; may contain plasmids that contribute to weediness	Anderson, 1952/Tables3 and e
*Proportion of total species*	Minute (several hundred species)	Not yet established (likely to be minute)	Hill, 1977/current article
*Association with human activity*	Evolution and ecology have been strongly favoured by land disturbance that creates and open (plant) habitats^i^	Evolution is likely to have occurred primarily in nature; but habitat dominance is commonplace in both natural and manmade environments	Anderson, 1952; Hill, 1977/Fig. 2; Table S1 and g
*Problematic for human endeavours*	Yes, due to loss of crop-plant yields^j^; toxicity/harm to livestock; role as hosts for crop pests and diseases, etc.	Yes, due to spoilage of foods, drinks; some species are pathogens of crop plants, livestock or humans; may emerge to take over industrial fermentations and other processes^k^	Hill, 1977/Table S1
*Value to humankind*	Disproportionately important (origin of many crop-plant species; plant weeds have food value for humans or livestock; model organisms for research)	Disproportionately important (key sources of bulk chemicals and antimicrobials^l^; for food and drinks production, and other biotechnological applications)	Hill, 1977; Koornneef and Meinke, 2010/; see *Concluding remarks*

a Typical traits.

b Plant weeds may not be the dominating plant species.

c Not necessarily the primary habitat of the microbial weed species.

d Vigour can result from a combination of traits such as fast growth, stress tolerance, and immunity to toxins and diseases (see Tables 2–6).

e Plant weeds typically have a high output of seeds under both favourable and poor growth-conditions, and can produce seed early (prior to maturity of the parent plant; Hill, 1977).

f Each microbial weed species may secrete diverse antimicrobial substances which can be the primary factor in habitat dominance (see Table 7; Hallsworth, 1998; Walker, 2011). For examples of the concentrations at which moderately chaotropic stressors, potent chaotropes and hydrophobic stressors can inhibit cellular systems see Bhaganna and colleagues (2010). Toxins such as antibiotics, and VOCs, biosurfactants, and other stressors can have additional key roles in the cellular biology and ecology of microbes.

g See Hallsworth and colleagues (2003a); Bhaganna and colleagues (2010).

h Some stressors of biotic origin are structurally identical to and/or exert the same stress mechanisms as stressors of abiotic origin (e.g. see Fig. 2; Tables 2–4 and 6).

i Agricultural practices and crop-plant life-cycle can also select for early seed production and other weed traits (Hill, 1977).

j Crop-yield losses caused by plant weeds are equivalent to those caused by plant pests or plant diseases (Cramer, 1967).

k Open (microbial) habitats are not only the starting point for food and drinks fermentations, but many foodstuffs themselves represent open habitats and it is frequently microbial weed species that cause spoilage and therefore determine shelf-life. Open habitats located on and within host organisms can be readily invaded by microbial weed species including those with pathogenic activities (Cerdeño-Tárraga, *et al*., 2005). This has given rise to the use of probiotics (Molly *et al*., 1996; Bron *et al*., 2012) and a practice of preserving the skin microflora in babies and infants by avoiding use of soaps or detergents (Capone *et al*., 2011).

l Antimicrobial substances are used for various applications; as biofuels, biocides, pharmaceuticals, flavour compounds, food preservatives, etc.

**Table 2:** Growth windows for *Pseudomonas putida* under habitat-relevant stresses*.*^a^

	Stressor concentration (mM) causing *P. putida* growth-rate inhibition of:			
Type of stressor	Specific examples	50%^b^	100%^b^	Notes and references
Hydrophobic compounds^c^	1,2,3-Trichlorobenzene^d^	0.20	0.40	*Pseudomonas putida* can be exposed to hydrophobic stressors in plant exudates, hydrocarbon-contaminated environments, and as hydrophobic catabolites and antimicrobials from *P. putida* or other microbes (see Table6; Timmis, 2009; Bhaganna *et al*., 2010)
	γ-Hexachlorocyclohexane^d^,^e^	0.00034	0.00072	
	2,5-Dichlorophenol^d^	0.70	0.90	
	n-hexane^d^	0.45	0.71	
	Toluene^d^	4.0	4.6	
	Benzene^d^	8.4	11.0	
Surfactants and aromatic solutes (highly chaotropic)	Tween® 80^f^,^g^	108	164	Cells come into contact with biosurfactants of microbial origin, and with chaotropic aromatics from the sources listed above (see also Hallsworth *et al*., 2003a; Timmis, 2009; Bhaganna *et al*., 2010)
	Phenol^h^	9.0	13.0	
	o-cresol^e^	3.82	8.10	
	Benzyl alcohol^h^	20.0	32.7	
	Sodium benzoate^e^	69.2	115	
Chaotropic salts	CaCl_2_^e^	210	302	Chaotropic salts occur in soils, sediments, evaporite deposits, the Dead Sea, brine lakes (including the Discovery Basin located beneath the Mediterranean Sea and the Don Juan Pond in the Antarctic) and marine-associated habitats including bitterns (see Table S1); can be highly stressful for, and lethal to, microbial systems (Hallsworth *et al*., 2003a; 2007; Duda *et al*., 2004)
	MgCl_2_^d^	182	311	
	Guanidine-HCl^h^	125	640	
	LiCl^h^	595	825^i^	
Alcohols and amides (moderately chaotropic)	Butanol^d^	158	208	Alcohols are produced by some species as antimicrobials (Table6); ethanol is used as a biocide and can occur as an anthropogenic pollutant. Urea may be produced as an antimicrobial by some bacteria (Table6) and is used as an agricultural fertilizer to which soil bacteria are exposed
	Ethanol^h^	600	993^i^	
	Urea^h^	650	950	
	Formamide^d^	168	1080^i^	
Relatively neutral substances	Methanol^d^	970	1440	Some organic compounds have little chao- or kosmotropic activity at biologically relevant concentrations. At low concentrations glycerol is relatively neutral but, despite its role as a stress protectant, can act as a chaotropic stressor at molar concentrations (Williams and Hallsworth, 2009)
	Ethylene glycol^h^	1300	2160^i^	
	Glucose^j^	840	1400	
	Glycerol^j^	770	2120^i^	
	Maltose^j^	326	1300	
Kosmotropic compatible solutes	Proline^j^	800	2000	The majority of microbial compatible solutes are kosmotropic, including other compatible solutes found in *P. putida* and other bacteria such as mannitol, betaine and ectoine. *Pseudomonas* may also come into contact with some of these compounds via exposure to plant exudates
	Sorbitol^j^	503	1200	
	Dimethyl sulfoxide^j^	353	1300	
	Trehalose^j^	474	771	
	Glycine^j^	99	370	
	Betaine^j^	1570	2560	
Kosmotropic salts	NaCl^j^	650	750	NaCl is environmentally ubiquitous, and is the dominant salt in most marine habitats including bioaerosols (Table S1); ammonium sulfate and KH_2_PO_4_ are commonly applied to soils as fertilizers. In many habitats kosmotropic salts are the primary osmotic stressors, but stresses imposed on the cell are determined by the net combination of solute activities of ions and other substances present (see Hallsworth *et al*., 2007)
	KH_2_PO_4_^j^	118	200	
	Ammonium sulfate^j^	198	350	
	Sodium citrate^j^	53	500	
Polysaccharides (kosmotropic)	Dextran 40000^j^	10	17	*Pseudomonas putida* is exposed to the kosmotropic activities of and/or matric stress induced by polysaccharides such as microbe- and plant-derived extracellular polymeric substances, humic substances, etc.
	Polyethylene glycol 3350^j^	43	120	
	Polyethylene glycol 6000^j^	20	40	

a Pertinent to the phyllosphere, rhizosphere, hydrocarbon-polluted environments, and other habitats (see Table S1), including catabolic products of hydrocarbon degradation, antimicrobial substances (see Table 6), compatible solutes, and other *P. putida* metabolites. At sufficient concentrations solutes such as glucose, maltose, proline, sorbitiol, glycine, betaine, NaCl and KH_2_PO_4_ induce osmotic stress; whereas dextran and high *M*_r_ polyethylene glycols induce matric stress (see Brown, 1990); hydrophobic substances typically induce a (chaotropicity-mediated) hydrocarbon-induced water stress (Bhaganna *et al*., 2010; Cray *et al*., 2013); and other aromatics, alcohols, amides and specific salts cause chaotrope-induced water stress (Hallsworth *et al*., 2003a; Cray *et al*., 2013).

b Relative to no-added stressor controls (see Hallsworth *et al*., 2003a).

c Log P > 1.95 (see Bhaganna *et al*., 2010; Cray *et al*., 2013).

d These data were taken from Bhaganna and colleagues (2010); *P. putida* KT2440 (DSMZ 6125) was grown in a minimal mineral-salt broth (with glucose and NH_4_Cl as the sole carbon and nitrogen substrates respectively; see Hartmans *et al*., 1989; Bhaganna *et al*., 2010) at 30°C. The media for control treatments had no stressors added. Some *P. putida* strains can tolerate benzene at up to 20 mM (Volkers *et al*., 2010).

e The pesticide γ-hexachlorocyclohexane (γ-HCH) is also known as lindane.

f *Pseudomonas putida* KT2440 was grown in modified Luria–Bertani broth at 30°C; stressors were incorporated into media prior to inoculation, and growth rates calculated, as described by Hallsworth and colleagues (2003a). The media for control treatments had no stressors added.

g Tween® 80 was used as a model substitute for biosurfactants (Cray *et al*., 2013).

h These data were taken from Hallsworth and colleagues (2003a); *P. putida* KT2440 was grown in modified Luria–Bertani broth at 30°C as described in footnote (f).

i Extrapolated values.

j Glycerol is a notable exception (Williams and Hallsworth, 2009).

**Table 3:** Stress tolerance and energy generation: cellular characteristics that contribute to weed-like behaviour.^a^

Metabolites, proteins, molecular characteristics, and other traits	Function(s)	Phenotypic traits	Notes and references
**Tolerances to multiple environmental stresses**			
Ability to select from an array of functionally diverse compatible solutes in response to specific stress parameters	Specific compatible solutes can protect against osmotic stress, temperature extremes, freezing, dehydration and rehydration, chaotropicity, and/or hydrophobic stressors (Crowe *et al*., 1984; Brown, 1990; Hallsworth *et al*., 2003b; Bhaganna *et al*., 2010; Chin *et al*., 2010; Bell *et al*, 2013)	Ability to maintain functionality of macromolecular systems and/or cell turgor under diverse stresses	Some microbes utilize a wide array of compatible solutes depending on the type and severity of stress; e.g. *Pseudomonas putida* is exceptional among bacteria in its ability to synthesize and accumulate protectants as diverse as betaine, proline, *N*-acetylglutaminylglutamine amide, glycerol, mannitol and trehalose (D'Souza-Ault *et al*., 1993; Kets *et al*., 1996; Bhaganna *et al*., 2010); *Saccharomyces cerevisiae* can accumulate proline, glycerol and trehalose (see Table6; Kaino and Takagi, 2008). Like other fungi, *Aspergillus* species utilize a range of polyols as well as trehalose (Hallsworth and Magan, 1994c; Hallsworth *et al*., 2003b) but their exceptional ability to generate energy under stress (see below) may aid glycerol retention and thereby extending the growth window at low water activity (Hocking, 1993; Williams and Hallsworth, 2009)
Upregulation of protein-stabilization proteins in combinations tailored to specific stresses^b^	In addition to stressors (including those of biotic origin; Table6), protein-stabilization proteins also protect against perturbations induced by stresses such as temperature extremes and desiccation (Arsène *et al*., 2000; Ferrer *et al*., 2003)	Maintenance of protein structure and function during environmental challenges	Several studies show highly efficient responses of *P. putida* to chaotropic and hydrophobic stressors via upregulation of between 10 and 20 protein-stabilizing proteins (Hallsworth *et al*., 2003a; Santos *et al*., 2004; Segura *et al*., 2005; Domínguez-Cuevas *et al*., 2006; Tsirogianni *et al*., 2006; Volkers *et al*., 2006; Ballerstedt *et al*., 2007). The PalA protein in *Aspergillus nidulans* induces a rapid upregulation of protein-stabilization proteins in response to extreme pH (Freitas *et al*., 2011); high production rates of protein-stabilization proteins enhance tolerance to chaotropic salts and alcohols in *S. cerevisiae* and *Lactobacillus plantarum*, to hydrophobic stressors in *S. cerevisiae* and *Escherichia coli* (for references, see Bhaganna *et al*., 2010), and low temperature, salt and other stresses in *Rhodotorula mucilaginosa* (Lahav *et al*., 2004)
Intracellular accumulation of ions for osmotic adjustment; intracellular proteins structurally adapted to high ionic strength	The utilization of ions for osmotic adjustment avoids the energy-expensive synthesis of organic compatible solutes (Kushner, 1978; Oren, 1999)	Ability to maintain metabolic activity at high extra- and intracellular ionic strength	*Haloquadratum walsbyi*, and *Salinibacter ruber* (unusually for a bacterium), utilize the so-called ‘salt-in’ strategy (Oren *et al.,* 2002; Bardavid and Oren, 2012; Saum *et al*., 2012)
Diverse proteins and pathways conferring tolerance to solvents, chaotropic solutes and hydrophobic stressors	Solvent-tolerant species such as *P. putida* can function at high concentrations of chaotropic and hydrophobic stressors due to the collective activities of solvent pumps that expel solvent molecules, pathways that catabolize the solvent, membrane-stabilization proteins (see below), compatible solutes (see above), and additional chaotrope- and solvent-stress responses (Ramos *et al*., 2002; Timmis, 2002; Hallsworth *et al*., 2003a; Volkers *et al*., 2006; Bhaganna *et al*., 2010; Fillet *et al*., 2012)	High tolerance to chaotropic and hydrophobic solvent stressors	Due to its exceptional solvent tolerance *P. putida* is the organism of choice for many industrial applications including biotransformation in two-phase systems and bioremediation (Timmis, 2002).
Membrane-stabilization proteins; e.g. *cis/trans*-isomerase (CTI)	Membrane stabilization under stress	Enhanced tolerance to stresses that impact membrane structure	Several proteins are involved in maintaining bilayer rigidity in *P. putida* during exposure to toluene and other solvents or hydrophobic stressors (Bernal *et al*., 2007)
Proteins involved in the moderation of membrane fluidity	Maintenance of membrane fluidity and membrane-associated processes under conditions such as low temperature (Turk *et al*., 2011)	Enhanced tolerance to diverse, physicochemical stress parameters	For example *Aureobasidium pullulans, Cryptococcus liquefaciens* and *R. mucilaginosa* (Turk *et al*., 2011)
Promoter(s) that simultaneously control multiple stress-response genes, e.g. stress-response elements (STREs)	Activation of multiple cellular stress-responses	Enhanced tolerance to multiple stress-parameters	The transcription factors (Msn2p and Msn4p) involved in STRE-mediated stress responses in *S. cerevisiae* accumulate in the nucleus upon heat-, ethanol- (chaotrope-), and osmotically induced stresses, and during nutrient limitation. The STRE is upstream of genes for protein-stabilization proteins (see above) metabolic enzymes, and cellular organization proteins (Görner *et al*., 1998; Moskvina *et al*., 1998)
Proteins involved in DNA protection under stress; e.g. DNA-binding protein (Dps)	Formation of a protein:chromosome complex that protects DNA	Enhanced DNA stability and reduced risk of mutation in relation to multi-farious stresses	The role of Dps has been well-characterized in *E. coli* in relation to diverse environmental challenges including reactive oxygen species, iron and copper toxicity, thermal stress and extremes of pH (Nair and Finkel, 2004)
Pathways for synthesis of carotenoids (e.g. β-carotene in *Dunaliella salina*; torularhodin in *Rhodotorula* spp.)	Carotenoids absorb light and thereby protect cellular macromolecules and organelles from exposure to ultraviolet	Tolerance of exposure to ultraviolet irradiation	Synthesis of β-carotene is upregulated in response to light in *D. salina* (Chen and Jiang, 2009); carotenoids are concentrated in the plasma membrane of *S. ruber* (Antón *et al*., 2002); studies of *R. mucilaginosa* show that torularhodin enhances tolerance to ultraviolet B exposure (Moliné *et al*., 2010)
Preferential utilization of a chaotropic compatible solute at high NaCl concentration and/or low temperature	Rigidification of macromolecular structures at high NaCl and low temperature can induce metabolic inhibition (Brown, 1990; Ferrer *et al*., 2003; Chin *et al*., 2010) that can be compensated for by glycerol and/or fructose (Brown, 1990; Chin *et al*., 2010)	Enhanced tolerance to extreme conditions that rigidify macromolecular systems	The high intracellular concentrations of glycerol in *D. salina* under salt stress and glycerol and/or fructose in and *Aspergillus* and *Eurotium* spp. and *R. mucilaginosa* at low temperature are able to extend growth windows under these extreme conditions (Brown, 1990; Lahav *et al.,* 2002; Chin *et al*., 2010). *Dunaliella salina* has membranes with reduced glycerol permeability thereby aiding retention of this compatible solute (Bardavid *et al*., 2008)
Efficient system to eliminate free radicals (see above)	Elimination of reactive oxygen species (see also Table5)	Various substances and stress parameters can induce oxidative stress	Diverse environmental substances and parameters induce oxidative stress, either directly or indirectly (e.g. via lipid peroxidation). For example, chaotropic stressors and heat shock have been associated with the induction of oxidative stress responses in *S. cerevisiae* and *P. putida* (Costa *et al*., 1997; Hallsworth *et al*., 2003a)
Proteins associated with regulation of cytosolic pH; e.g. the lysine decarboxylase system (coded for by the *cadBA* operon); the Na^+^/H^+^ antiporter	Conversion and export of lysine/cadaverine that results in increased pH of the cytosol in bacteria (Álvarez-Ordóñez *et al*., 2011); the Na^+^/H^+^ antiporter Nha1 decreases intracellular hydrogen ion concentration	Tolerance of low-pH environments	Decarboxylase systems that increase intracellular pH also exist for other amino acids (Álvarez-Ordóñez *et al*., 2011); Nha1 greatly enhances viability of *Saccharomyces boulardii* at pH 2 (dos Santos Sant'Ana *et al*., 2009)
Multidrug efflux pumps (for details see Table6)			
Enzymes for synthesis of extracellular polymeric substances	Production and secretion of polymeric substances	Enhanced tolerance to multiple stressors and stress parameters	Extracellular polymeric substances have been shown to confer protection to *E. coli* cells exposed to high temperature, low pH, salt and oxidative stress (Chen *et al*., 2004), and are associated with stress protection in *Clostridium* spp., *Pantoea ananatis*, *P. putida* and other *Pseudomonas* spp. (Chang *et al*., 2007; Morohoshi *et al*., 2011; see also Table 6)
Upregulation of proteins involved in protein synthesis under stress	To enhance or maintain the quantity, type, and functionality of cellular proteins under stress	Maintenance of the cellular system and its metabolic activity under stress	Can be upregulated in response to stress induced by high temperature, chaotropic solutes, hydrophobic stressors and other stress parameters (e.g. *P. putida*; Hallsworth *et al*., 2003a; Volkers *et al*., 2009)
Polyploidy, aneuploidy, association with plasmids, and/or hybridization that enhance(s) stress tolerance	Whereas hybridization, polyploidy, aneuploidy and association with plasmids can be ecologically disadvantageous, such traits can enhance the vigour, stress tolerance and competitive ability of some microbes such as *Pichia* spp., *S. cerevisiae, P. putida* and *S. ruber* (Table1; Rainieri *et al*., 1999; Naumov *et al*., 2001; Mongodin *et al*., 2005; Tark *et al*., 2005; Dhar *et al*., 2011; Chen *et al*., 2012)	Enhanced stress tolerance	Aneuploid cells of *S. cerevisiae* have enhanced tolerance to oxidative stress and diverse inhibitors of biochemical processes (Chen *et al*., 2012); polyploidy can enhance vigour and stress tolerance in some species (see also Tables1 and c); the stress tolerance of some microbial weeds is enhanced by their association with plasmids (e.g. the *P. putida* tol-plasmid that enhances solvent tolerance; Tark *et al*., 2005; Domínguez-Cuevas *et al*., 2006; the *S. ruber* plasmid contains a gene involved in ultraviolet protection; Mongodin *et al*., 2005)
Polyextremophilic growth-phenotype	Ability to grow optimally under multiple, extreme conditions; e.g. extremely acid and alkali (pH 2–10), high-salt (2.5 M NaCl), and low-temperature conditions (Lahav *et al.,* 2002; Libkind and Sampaio, 2010)	Polyextremophile	*Rhodotorula mucilaginosa* is capable of growth over an incredible range of physicochemical conditions (including almost the entire pH range for microbial life) and is able to colonize a correspondingly wide diversity of environments (for examples see main text)
Genes associated with salt stress	Sugar catabolism (e.g. *RmPGM2*, *RmGPA2*, *RmACK1*, *RmMCP1* and *RmPET9*), protein glycosylation (e.g. *RmSEC53*), aromatic amino acid synthesis (e.g. *RmARO4*), regulation of protein synthesis (e.g. *RmANB1*)	Maintenance of protein synthesis and energy generation under salt stress	Between 10 and 20 genes are involved in increased tolerance to NaCl and/or LiCl in *R. mucilaginosa* (Lahav *et al*., 2004; Gostinčar *et al*., 2012)
Metabolites associated with resistance to antimicrobial plant metabolites and survival in phyllosphere habitats	Metabolites involved in quorum sensing may enhance competitive ability on the leaf surface	Enhanced survival and competitive ability	By contrast to other phyllosphere microbes, *P. ananatis* is resistant to the inhibitory effects of plant alkaloids and this correlates with the production of metabolites involved in quorum sensing such as *N*-acyl-homoserine lactone (Enya *et al*., 2007)
**High-efficiency energy-generation systems**			
Light-activated proton pumps (e.g. xanthorhodopsin)	To generate a proton-motive force, utilizing light as an energy source	Utilization of light for transmembrane proton transport	Xanthorhodopsin, found in *S. ruber*, is a proton pump made up of two chromophores; a light-absorbing carotenoid and a protein (Balashov *et al*., 2005); a light-activated proton pump was also recently described in *H. walsbyi* (Lobasso *et al*., 2012)
High expression of genes involved in energy generation (e.g. *fba*, *pykF*, *atpA* and *atpD*^c^)	Rapid catabolism of carbon substrates leading to enhanced synthesis of ATP	Efficient energy-generation	High expression of *E. coli* genes *fba*, *pykF*, *atpA* and *atpD* can be associated with ability to grow rapidly, enhanced stress tolerance and – by implication – greater competitive ability (Karlin *et al*., 2001)
Enhanced energy-efficiency at low nutrient concentrations (e.g. see glutamate dehydrogenase entry in Table5)			
Upregulation of proteins involved in energy generation under chaotropic and solvent-induced (hydrocarbon) stresses (Hallsworth *et al*., 2003a; Volkers *et al*., 2006).	Maintenance of energy generation close to the point of system failure under hostile conditions	Efficient energy generation during potentially lethal challenges	The exceptional ability of *P. putida* to respond and adapt to chaotropic, solvent and hydrocarbon stressors has, in part, been attributed to considerable increases in energy- and NAD(P)H-generating systems
Idiosyncratic genome modifications associated with reinforced energy metabolism	Enhanced primary metabolism and energy-generation systems	Enhanced vigour and energy production	*Aspergillus* species have numerous genome modifications, such as gene duplications for pyruvate dehydrogenase, citrate synthase, aconitase and malate dehydrogenase that enhance primary metabolism including glycolysis and the TCA cycle (Flipphi *et al*., 2009)
Efficient utilization of intracellular reserves for energy generation	Catabolism of stored substances to boost growth, stress tolerance, production of secondary metabolites, motility and/or other key cellular functions under stress	Ability to maintain energy production during nutrient limitation and under hostile conditions	Efficient storage (see Table5) and utilization of energy-generating substances such as trehalose, glycogen and polyhydroxyalkanoates confers a competitive advantage in *S. cerevisiae* and other species (Castrol *et al*., 1999; Kadouri *et al*., 2005)
Concomitant energy generation and synthesis of antimicrobials with no loss of ATP-generation efficiency	Via aerobic fermentation *S. cerevisiae* can simultaneously generate energy while synthesizing the chaotropic stressor ethanol (see Table6)	Energy generation linked to synthesis of an antimicrobial	Each glucose molecule fermented by *S. cerevisiae* produces two ethanol molecules and two molecules of ATP (Piškur *et al*., 2006)

a The current table provides examples of proteins, genes or other characteristics that can be associated with and give rise to weediness; the categories of traits are not mutually exclusive. Individual traits may not be unique to microbial weeds; however, weeds species are likely to have a number of these types of characteristics.

b For example chaperonins, heat-shock proteins and cold-shock proteins.

c That code for fructose-1,6-*bis*phosphate aldolase, pyruvate kinase and ATP synthase subunits F1 α and F1 β respectively.

This article focuses on the following questions: (i) what types of substrate or environment facilitate the emergence of dominant microbial species, (ii) are there archetypal weed species that can consistently dominate microbial communities, (iii) what are the stress biology, nutritional strategies, energy-generating capabilities, antimicrobial activities and other competitive strategies of microbial weeds, (iv) which components and characteristics of microbial cells form the mechanics of weed biology, (v) what are the properties of open (microbial) habitats that promote the emergence of weed species, and (vi) how are microbial weeds conceptually distinct from plant weeds, and other ecophysiological groups of microorganism?

### Dominance within microbial communities

The microbial diversity of specific habitats, as well as that of Earth's entire biosphere, is an area of intensive scientific interest that has implications within the fundamental sciences and for drug discovery, biotechnology and environmental sustainability. Biodiversity provides the material basis for competition between microbial taxa, species succession in communities, and other aspects of microbial ecology that collectively impact the evolution of microbial species (Cordero *et al*., 2012). We believe that certain microbes are genetically, and thus, metabolically hard-wired to dominate communities (monopolizing available resources and space) and thereby reduce biodiversity within the habitat. Microbial communities can reach a stationary-phase or climax condition with a relatively balanced species composition (McArthur, 2006). However whether tens or thousands of microbial species are initially present (Newton *et al*., 2006) the community can become dominated by a single – or two to three – species (Fig. 1; Table S1; Randazzo *et al*., 2010; Cabrol *et al*., 2012). For example the indigenous microflora of olives is comprised of significant quantities of phylogenetically diverse species such as *Aureobasidium pullulans*, *Candida* spp., *Debaryomyces hansenii*, *Metschnikowia pulcherrima*, *Pichia guilliermondii*, *Pichia manshurica*, *Rhodotorula mucilaginosa* and *Zygowilliopsis californica* (Fig. 1Bi; Nisiotou *et al*., 2010). Nisiotou and colleagues (2010) found that species succession and the stationary-phase community during olive fermentations are determined by one dominating genus, *Pichia*, three species of which account for almost 100% of the community by 35 days (see Fig. 1Biii). The most prevalent of these, *Pichia anomala*, is a microbial generalist that is not only able to inhabit ecologically and nutritionally distinct environments (e.g. palm sugar, cereals, silage, oil-contaminated soils, insects, skin, various marine habitats; see Walker, 2011) but can also dominate the communities that develop in diverse habitat types (Table S1; Tamang, 2010; Walker, 2011). Studies of Pecorino Crotonese cheese fermentations reveal that, even though the curd contains > 300 bacterial strains, the community is eventually dominated by *Lactobacillus rhamnosus* (Randazzo *et al*., 2010).

Species that are able to dominate communities that develop in open habitats are not restricted to copiotrophs, generalists or any established ecophysiological grouping of microorganism (Table S1). Furthermore dominating species span the Kingdoms of life and, collectively, can be observed in most segments of the biosphere; from bioaerosols to solar salterns, oceans and freshwater to sediments and soils, the phyllosphere and rhizosphere, to those with molar concentrations of salts or sugars (see Table S1). The phenomenon can also be observed during food-production, food-spoilage and waste-decomposition processes where resource-rich habitats are available for colonization (see Table S1). The materials present in and the microbes living on these substrates are ultimately derived from the natural environment so community successions resulting in single-species dominance are likely to be commonplace in comparable open habitats within natural ecosystems (Table S1; Senthilkumar *et al*., 1993; Nisiotou *et al*., 2007; Reynisson *et al*., 2009; Rajala *et al*., 2011). Microbial weed species are able to dominate open habitats that progress to a closed condition (e.g. fermenting fruit juice) as well as those that remain in a more or less perpetually open state (e.g. the surface-fluid film of sphagnum moss and rhizosphere of plants growing in moist soils); see Table S1. Weeds emerge in habitats where competing cells are in close proximity (such as biofilms; Table S1; Rao *et al*., 2005) as well as those where there is limited mechanical contact between cells, such as the aqueous habitats of planktonic species (Table S1; Collado-Fabbri *et al*., 2011; Trigal *et al*., 2011; Leão *et al*., 2012); and in both extreme and non-extreme environments (Table S1). Some microbes emerge as a/the dominating species in diverse types of habitat including specialists such as *Lactobacillus* (that do so, in part, by acidifying their environment) and generalists such as *Aspergillus*, *Pichia* and *Pseudomonas* species (Table S1; Steinkraus, 1983; Tamang, 2010). For weed species such as *Saccharomyces cerevisiae* (high-sugar environments), *Haloquadratum walsbyi* (aerobic, hypersaline brines), and *Gonyostomum semen* and *Microcystis aeruginosa* (eutrophic freshwater) ability to dominate can be restricted to a specific type of habitat. Most of these microbes do not populate their respective habitat as a one-off or chance event; they can consistently dominate their microbial communities and do so regardless of their initial cell number (Table S1; for *P. anomala* see Fig. 1; for *S. cerevisiae* see Pretorius, 2000; Renouf *et al*., 2005; Raspor *et al*., 2006; for *L. rhamnosus* see Randazzo *et al*., 2010). It is therefore intriguing to consider what combination(s) of traits can give rise to a weed phenotype.

## Cellular biology of microbial weeds

### Stress tolerance and energy generation

During habitat colonization, even in apparently stable, homogenous environments such as sugar-based solutions, there are dynamic/drastic changes in stressor concentrations, antimicrobials and stress parameters (D'Amore *et al*., 1990; Hallsworth, 1998; Eshkol *et al*., 2009; Fialho *et al*., 2011; see also below). As a result of these fluctuations microbes are exposed to multiple stresses some of which present potentially lethal challenges to the cell. In soil and sediments, for example the water activit can oscillate from low to high and high to low; these substrates typically cycle between desiccation and rehydration and are subjected to changes in solar radiation, temperature, and even freezing and thawing. Soils and sediments can be physically heterogeneous such that water availability, solute concentrations and other stress parameters exhibit profound spatial fluctuations. In addition, intracellular metabolites and extracellular substances (of both biotic and abiotic origin) impose stresses due to ionic, osmotic, chaotropic, hydrophobic and other activities of solutes (see Brown, 1990; Hallsworth *et al*., 2003a; 2007; Lo Nostro *et al*., 2005; Bhaganna *et al*., 2010; Chin *et al*., 2010). On the one hand resilience to stress undoubtedly contributes to competitive ability but on the other a vigorous growth phenotype and reinforced stress biology also require extraordinary levels of energy generation. Furthermore, microbes that can achieve a greater net gain in energy than competing species will have an ecological advantage (Pfeiffer *et al*., 2001). Here we suggest that species able to dominate microbial communities are exceptionally well equipped to resist the effects of multiple stress parameters, and that many have high-efficiency energy-generation systems.

We tested the hypothesis that microbial weeds are exceptionally tolerant to habitat-relevant stresses for two species; the soil bacterium *Pseudomonas putida* (Table 2) and the specialist yeast *S. cerevisiae* (see below). Environments in which *P. putida* is a major ecological player (Table S1) typically contain an array of chemically diverse aromatics and hydrocarbons that induce chaotropicity-mediated stresses (Hallsworth *et al*., 2003a; Bhaganna *et al*., 2010; Cray *et al*., 2013). This bacterium is not only renowned for its metabolic versatility, but is also known for its tolerance to specific solutes and solvents (Hallsworth *et al*., 2003a; Domínguez-Cuevas *et al*., 2006; Bhaganna *et al*., 2010). We compared the biotic windows for growth of *P. putida* for eight mechanistically distinct classes of stress using 37 habitat-relevant substances (Table 2). This bacterium was able to tolerate all substances within each group of stressor; this is the first data set to demonstrate that a mesophilic bacterium can tolerate the following combination of stresses: up to 4–11 mM hexane, toluene and benzene; 8–115 mM concentrations of aromatic, chaotropic solutes; 300–825 mM chaotropic salts; 1300–2160 mM sugars or polyols; 200–750 mM kosmotropic salts; and considerable levels of matric stress (Brown, 1990) generated by kosmotropic polysaccharides (Table 2). The *P. putida* tolerance limits to highly chaotropic substances and hydrophobic compounds were superior to those of other microbes (including extremophiles and polyextremophiles; Table 2; Hallsworth *et al.,* 2007; Bhaganna *et al*., 2010). Whereas prokaryotes are generally less tolerant to solute stress than eukaryotes (Brown, 1990), the resilience to this array of mechanistically diverse solute stresses (Table 2) has neither been equalled nor been surpassed by any microbial species to our knowledge.

*Pseudomonas putida* is able to avoid contact with or prevent accumulation of stressors by using solvent-efflux pumps and synthesizing extracellular polymeric substances to form a protective barrier (Table 3). This bacterium also utilizes highly efficient macromolecule-protection systems that are upregulated in response to multiple environmental stresses including between 10 and 20 protein-stabilization proteins, oxidative-stress responses, and upregulation of energy generation and overall protein synthesis (Table 3). Furthermore, *P. putida* can synthesize an array of compatible solutes including betaine, proline, *N*-acetylglutaminylglutamine amide, glycerol, mannitol and trehalose (Table 3). Individual compatible solutes are unique in their physicochemical properties, interactions with macromolecular systems and functional efficacy for specific roles within the cell (e.g. see Chirife *et al*., 1984; Crowe *et al*., 1984; Brown, 1990; Hallsworth and Magan, 1994a; 1995; Yancey, 2005; Bhaganna *et al*., 2010; Bell *et al*., 2013). Microbes that are able to selectively utilize/deploy any substance(s) from a range of diverse compatible solutes have enhanced tolerance to diverse stresses (e.g. Hallsworth and Magan, 1995; Bhaganna *et al*., 2010). The range of compatible solutes utilized by *P. putida* may be unique for a bacterium (see Brown, 1990) and can confer tolerance to diverse stresses including those imposed by chaotropes and hydrocarbons (Bhaganna *et al*., 2010).

The exceptionally wide growth-windows of *Aspergillus* species in relation to a number of stress parameters correlate with their ability to dominate communities in both extreme and non-extreme (but physicochemically dynamic) environments including soils, plant necromass, saline and very high-sugar habitats (Table S1). At least 20 species of *Aspergillus* and the closely related genus *Eurotium* are xerotolerant (Pitt, 1975; Brown, 1990). *Aspergillus penicillioides* is capable of hyphal growth and conidial germination down to water activities of 0.647 and 0.68 respectively (Pitt and Hocking, 1977; Williams and Hallsworth, 2009); *Aspergillus echinulatus* can germinate down to 0.62 (Snow, 1949). Halotolerant species such as *Aspergillus wentii* can grow on the highly chaotropic salt guanidine-HCl at a higher concentration than any other microbe; up to 930 mM (≡ 26.5 kJ g^−1^ chaotropic activity; Hallsworth *et al*., 2007) and, along with *Aspergillus oryzae*, up to ∼ 4 M NaCl or KCl (Hallsworth *et al*., 1998). *Aspergillus* (and *Eurotium*) strains are capable of reasonable growth rates close to 0°C (Chin *et al*., 2010), and some reports suggests that the biotic windows for some *Aspergillus* species ultimately fail at ≤ −18°C (Vallentyne, 1963; Siegel and Speitel, 1976). Whereas a number of comparable fungal genera show moderate levels of stress tolerance, *Aspergillus* species generally out-perform their competitors and top numerous league tables for ascomycete stress tolerance (see Pitt, 1975; Wheeler and Hocking, 1993; Williams and Hallsworth, 2009).

Studies of seven *Aspergillus* species have revealed a number of genome modifications within this genus that reinforce both primary metabolism and energy generation (Table 3; Flipphi *et al*., 2009). These include gene duplications for key enzymes that control metabolic flux, such as pyruvate dehydrogenase, citrate synthase, aconitase, and malate dehydrogenase that enhance glycolysis and the TCA cycle (Flipphi *et al*., 2009). There is evidence that, at the low water activities (high osmotic pressures) associated with very high-salt and high-sugar habitats, the biotic window of xerophilic fungi fail due to the prohibitive energy expenditure required to prevent the high levels of intracellular glycerol from leaking out of the membrane (Hocking, 1993). The adaptability and competitive ability of *Aspergillus* species (most of which can also proliferate under non-extreme conditions) may be attributed, in part, from enhanced energy-generating capability. *Aspergillus* species (like many fungi) can utilize a range of polyols – as well as trehalose – as compatible solutes based on their individual strengths as protectants against osmotic stress, desiccation rehydration, chaotropes etc. (Crowe *et al*., 1984; Brown, 1990; Hallsworth *et al*., 2003b). *Aspergillus* and *Eurotium* strains can also accumulate chaotropic sugars from the medium, or preferentially synthesize and accumulate chaotropic glycerol, in order to enhance growth at temperatures below ∼ 10°C (Chin *et al*., 2010; see also below).

*Salinibacter ruber* is a halophilic bacterium found in high numbers at molar salt concentrations in aerobic aquatic habitats that are otherwise more densely populated by archaeal than bacterial species. Unusually for a bacterium, *S. ruber* takes in ions to use as compatible solutes and thereby avoids synthesis of organic compatible solutes that would be energetically unfavourable in environments of > 1 M NaCl (Oren, 1999) and has evolved intracellular proteins that are both structurally stable and catalytically functional in the cytosol at high ionic strength (Table 3). *Salinibacter ruber* (as well as the alga *Dunaliella salina* and yeast *R. mucilaginosa*) accumulates carotenoids in response to light which can protect against oxidative stress that is a potential hazard in salterns exposed to high levels of ultraviolet (Table 3). *Haloquadratum walsbyi*, an Archaeon that is also a dominant player in saltern communities (Table S1), also uses the ‘salt-in’ compatible-solute strategy, and – along with *S. ruber –* has light-activated protein pumps that generate proton-motive force and thereby boost energy generation (Table 3). In addition, *H. walsbyi* is unusually tolerant to the chaotropic salt MgCl_2_ that can reach high concentrations in evaporate ponds (Hallsworth *et al*., 2007; Bardavid *et al*., 2008). *Dunaliella salina* is also highly prevalent in saline habitats that (unusually for an alga) can successfully compete with the multitude of halophilic Archaea found in 5 M NaCl environments (see later; Table S1). This alga preferentially utilizes glycerol as a compatible solute, is able to accumulate glycerol to molar concentrations (6–7 M; see Bardavid *et al*., 2008), and has low membrane permeability to this compatible-solute thus aiding retention (Bardavid *et al*., 2008).

Glycerol is unique in its ability to maintain the flexibility of macromolecular structures under conditions that would otherwise cause excessive rigidification, e.g. molar concentrations of NaCl, or low temperature (Back *et al*., 1979; Hallsworth *et al*., 2007; Williams and Hallsworth, 2009; Chin *et al*., 2010). Species able to grow at low or subzero temperatures (e.g. *Aspergillus, Cryptococcus, Rhodotorula* species, including *R. mucilaginosa*) either primarily use glycerol as a compatible solute or preferentially accumulate this chaotrope at low temperature (Table 3; Lahav *et al*., 2004; Chin *et al*., 2010). *Rhodotorula mucilaginosa* is able to colonize – and frequently dominates – diverse substrates/environments including saline and non-saline soils, aquatic habitats (both liquid- and ice-associated), the acidic surface-film of sphagnum moss (as well as other plant tissues) and various surfaces within the human body and other animals (Table S1; Egan *et al.,* 2002; Trindade *et al*., 2002; Vital *et al*., 2002; Lahav *et al*., 2002; 2004; Lisichkina *et al*., 2003; Buzzini *et al*., 2005; Perniola *et al*., 2006; Goyal *et al*., 2008; Turk *et al*., 2011; Kachalkin and Yurkov, 2012). This ability is coupled with an exceptional level of tolerance to multiple stresses: *R*. *mucilaginosa* is (highly unusual for a yeast) a polyextremophile that is psychrophilic, highly salt-tolerant, and can growth over the majority of the pH range known to support microbial life (i.e. < 2 and > 10, see Table 3; Lahav *et al*., 2002).

Some microbial habitats such as fruits that have been disconnected from the vascular system (and immune responses) of the parent plant have a favourable water activity and high nutrient availability and are therefore potentially habitable by a wide range of microbes (Ragaert *et al*., 2006). As the microbial community develops on a detached fruit (or in apple or grape musts; see Table S1) there can be changes in the composition and concentration of sugars, osmolarity, water activity, ethanol concentration (and that of other antimicrobials), pH and other environmental factors that are severe enough to inhibit or eliminate most members of the original microflora (see Brown, 1990; Hallsworth, 1998; de Pina and Hogg, 1999; Pretorius, 2000; Bhaganna *et al*., 2010). The yeast *S. cerevisiae* that is relatively scarce in natural habitats (and even when present forms a quantitatively insignificant component of the natural community; Renouf *et al*., 2005; Raspor *et al*., 2006) is unsurpassed in its ability to emerge to dominate high-sugar habitats at water activities above 0.9 (Table S1; Hallsworth, 1998; Pretorius, 2000). We therefore compared its tolerance towards a range of habitat-relevant stressors with those of 12 other yeasts that occur at the insect–flower interface, on plant tissues, and/or are environmentally widespread (Fig. 2; Table 4). This experiment revealed that *S. cerevisiae* has a more vigorous growth phenotype and more robust stress tolerance than the other species tested with the exception of two species of *Pichia*, another genus characterized by weed-like traits (see Fig. 2; Table 4). Stress phenotypes of the yeasts assayed were compared on the basis of stressor type and mechanistically distinct stress-parameters in order to assess resilience to both the degree and diversity of environmental challenges (Table 4). Under the assay conditions it was the three *S. cerevisiae* strains, *Pichia (Kodamaea) ohmeri* and *Pichia sydowiorum* that exhibited the greatest tolerance to the widest range of solute-imposed stresses (Table 4). Other species studied were slow growers, have specific environmental requirements (e.g. the halotolerant *D. hansenii* and *Hortaea werneckii*), and/or otherwise lacked the capacity for vigorous growth on the high-sugar substrate at 30°C (see Fig. 2). Generally *S. cerevisiae* strains were fast-growing, xerotolerant, and highly resistant to chaotrope-induced, osmotic, matric and kosmotrope-induced stresses and are known to be acidotolerant and capable of growth over a wide temperature range, from ∼0°C to 45°C (Fig. 2A and B; Table 4). Although *Pichia* species can produce ethanol (*P. anomala* can produce up to 0.36 M ethanol; Djelal *et al*., 2005) when growing side by side in high-sugar habitats with *S. cerevisiae*, *Pichia* species are unable to tolerate the molar ethanol concentrations that *S. cerevisiae* can produce (Lee *et al*., 2011).

**Table 4:** Stress phenotypes and ecophysiological profiles of *Saccharomyces cerevisiae* relative to those of other yeast species

	Tolerance to diverse stressors^b^					Tolerance to distinct stress mechanisms^b^						
Yeast species^a^	Alcohols	Glycerol	Sugars	Salts	Others	Chaotropic	Osmotic	Matric	Kosmotropic	Additional notes on stress and growth phenotypes^c^	Typical habitat	Ecophysiological description^d^
***Saccharomyces cerevisiae* strain**												
CCY-21-4-13	Higher	Mid	Higher	Higher	Higher	Higher	Higher	Higher	Higher	T_opt_ 30–35°C; T_min_ ∼0.5°C; T_max_ 45°C pH_opt_ 3–7; pH_min_ ∼2.5^e^; fast grower	Fruits; other fleshy plant tissues; nectar	Multiple stress tolerances including sugar and ethanol^f^; **stress phenotype of a xerotolerant, acidotolerant, vigorous weed**
Alcotec 8	Higher	Mid	Mid	Mid	Higher	Higher	Mid	Higher	Mid	As above	As above	As above
CBS 6412	Higher	Mid	Higher	Mid	Higher	Higher	Mid	Higher	Mid	As above	As above	As above
**Other species (strain)**												
*Candida etchellsii* (UWOPS 01–168.3)	Lower	Higher	Mid	Higher	Mixed	Higher	Higher	Mid	Mid	Slow grower^g^	Pollinating insects; flowers; fermented foods	Intolerant to specific stressors; slow-growing
*Cryptococcus terreus* (PB4)	Mid	Mid	Higher	Mid	Higher	Mid	Mid	Higher	Mid	T_opt_ 10°C; T_min_ 1°C^h^; slow grower	Soils and sediments; subsurface environments	A slow-growing psychrophile^i^
*Debaryomyces hansenii* (UWOPS 05–230.3)	Mid	Mid	Higher	Higher	Higher	Mid	Mid	Higher	Lower	T_opt_ 30–32°C; pH_opt_ 5.5^j^; fast grower	High-salt environments and foods	Halotolerant; a fast-growing high-salt specialist
*Hansenula* (*Ogataea*) *polymorpha* (CBS 4732)	Higher	Mid	Mid	Mid	Higher	Higher	Lower	Higher	Higher	T_opt_ 37–43°C; pH_opt_ 4.5–5.5^h^	Soil, insect gut, fruit juice	Neither fast-growing nor highly stress tolerant; a high-temperature specialist
*Hortaea werneckii* (MZKI B736)	Mid	Mid	Higher	Higher^j^	Lower	Mid	Mid	Lower	Mid	T_opt_ ∼29°C^h^; slow grower	Salty environments; animal and plant surfaces	Intolerance to matric stress; a slow-growing halophile
*Kluyveromyces marxianus* (CBS 712)	Higher	Lower	Mid	Lower	Higher	Mid	Lower	Higher	Lower	T_opt_ ∼38–40°C; T_max_ 65°C^h^; not fast-growing	Milk; plant surfaces; natural fermentations	Thermotolerant, solute-intolerant and xero-intolerant^j^
*Pichia (Kodamaea) ohmeri* (UWOPS 05–228.2)	Higher	Mid	Higher	Higher	Higher	Higher	Higher	Higher	Mid	T_opt_ ∼33°C; T_max_ > 50°C^h^; fast- grower	Beetles; fruits and other plant	Multiple stress tolerances including ethanol and sugar; **stress phenotype of a xerotolerant, vigorous weed**
*Pichia* (*Komagataella*) *pastoris* (CBS 704)	Mid	Lower	Lower	Mid	Higher	Mid	Lower	Higher	Mid	pH_min_ ∼2.2^h^; not fast-growing	Rotting wood; slimes	Intolerant to osmotic stress, glycerol and sugar; a xero-intolerant yeast
*Pichia sydowiorum* (UWOPS 03–414.2)	Higher	Mid	Higher	Higher	Higher	Higher	Mid	Higher	Higher	pH_opt_ ∼6–8^h^; fast grower	Polluted environments; waste water	Multiple stress tolerances including ethanol and sugar; **stress phenotype of a xerotolerant, vigorous weed**
*Rhodotorula creatinivora* (PB7)	Lower	Lower	Mid	Mid	Lower	Lower	Lower	Mid	Higher	T_opt_ ∼10°C; T_min_ ∼1°C^h^; slow grower	Subsurface; soil	Intolerant to alcohols, glycerol; xerotolerant a psychrophilic slow grower
*Saccharomycodes ludwigii* (UWOPS 92–218.4)	Lower	Mid	Higher	Lower	Lower	Lower	Lower	Lower	Lower	pH_min_ ∼2^h^; slow grower	Grapes; plant tissues and fermentations	Intolerant to glycerol and NaCl; tolerant to sugars; an osmophile
*Zygosaccharomyces rouxii* (CBS 732)	Mid	Higher	Higher	Lower	Mixed	Higher	Mid	Mid	Mid	pH_opt_ 3–7; pH_min_ 1.5–2^h^; slow grower	Fruits and other high-sugar habitats	Intolerant to NaCl; tolerant to glycerol and sugars; an osmophile

a Sources of strains are given in Fig. 2.

b Based on data for growth on MYPiA at 30°C (see Fig. 2). Alcohols were ethanol and methanol, sugars were fructose and sucrose, salts were NaCl, MgCl_2_ and ammonium sulfate, the other stressors were proline, xantham gum and PEG 8000 (Fig. 2). Chaotrope stress is induced by ethanol, fructose, glycerol and MgCl_2_; osmotic stress by fructose, sucrose, NaCl and proline; matric stress by xantham gum and PEG 8000; and kosmotrope stress by ammonium sulfate (Fig. 2; Brown, 1990; Hallsworth, 1998; Hallsworth *et al*., 2003a; Hallsworth *et al*., 2007; Williams and Hallsworth, 2009; Bhaganna *et al*., 2010; Chin *et al*., 2010; Cray *et al*., 2013). Stress-tolerance assessments (higher, mid-range, lower) are relative to the range of those of the other species assayed, and based on values in Fig. 2.

c Temperature optima, minima and/or maxima (T_opt_, T_min_, T_max_) and pH optima, minima and/or maxima (pH_opt_, pH_min_, pH_max_) for growth were obtained from cited publications. Extreme growth phenotypes (fast or slow growers) were listed according to growth rates on MYPiA at 30°C (see also Fig. 2). Species designated as *slow grower*s did not have a vigorous growth phenotype under these conditions; i.e. all strains listed are able to grow rapidly within the assay period on/at their favoured media/temperature. The designation *slow growing* did not relate to an extended lag phase (data not shown).

d Primarily based on data presented in the current table as well as Fig. 2 (unless otherwise stated) so is most pertinent to high-sugar habitats at ∼30°C. Habitat dominance will ultimately be influenced by antimicrobial activities of species present within a community; see Table 6). Ecophysiological descriptions of weed-like species are underlined in bold.

e Values for temperature- and pH-tolerances were taken from: (for *S. cerevisiae*) Praphailong and Fleet (1997), Yalcin and Ozbas (2008) and Salvadó and colleagues (2011); (for *C. terreus*) Krallish and colleagues (2006); (for *D. hansenii*) Domínguez and colleagues (1997); (for *H. polymorpha*) Levine and Cooney (1973); (for *H. werneckii*) Díaz Muñoz and Montalvo-Rodríguez (2005); (for *K. marxianus*) Aziz and colleagues (2009); Salvadó and colleagues (2011); (for *P. ohmeri*) Zhu and colleagues (2010); (for *P. pastoris*) Chiruvolu *et al*. (1998); (for *P. sydowiorum*) Kanekar and colleagues (2009); (for *R. creatinivora*) Krallish and colleagues (2006); (for *S. ludwigii*) Stratford (2006); and (for *Z. rouxii*) Restaino and colleagues (1983) and Praphailong and Fleet (1997). Tolerance windows for *Candida etchellsii* are yet to be established.

f Under some conditions strains of *S. cerevisiae* can tolerate up to 28% v/v (20% w/v) ethanol (see Hallsworth, 1998).

g See also Krallish and colleagues (2006).

h In high-salt habitats, *H. werneckii* can grow at molar concentrations of NaCl (see Gunde-Cimerman *et al*., 2000) but was less salt tolerant under the conditions of this assay.

i This species appears to have a requirement for high water activity.

j Habitat dominance can also depend on factors such as production of antimicrobials (see also Table 6). *Pichia ohmeri* can dominate some habitats, *Pichia anomala* may be more vigorous as a weed species (see Rosa *et al*., 1990; Lee *et al*., 2011).

*Saccharomyces cerevisiae* is metabolically wired to concomitantly synthesize an antimicrobial chaotrope (ethanol), accumulate a compatible solute (glycerol) that affords protection against this chaotrope (see below), and at the same time generate energy without any loss of ATP-generation efficiency (Table 3): a remarkable phenotype that is coupled with the deployment of other compatible solutes under specific conditions (trehalose and proline). Trehalose, which can reach concentrations of up to 13% dry weight in *S. cerevisiae* (Aranda *et al*., 2004), is highly effective at stabilizing macromolecular structures that are exposed to chaotropic substances (e.g. ethanol, 2-phenylethanol) and enabling cells to survive desiccation–rehydration cycles (Crowe *et al*., 1984; Mansure *et al*., 1994). The highest reported tolerance to ethanol stress was in *S. cerevisiae* cells capable of growth and metabolism up to 28% v/v (≡ 20% w/v or 4.3 M; chaotropic activity = 25.3 kJ g^−1^; Hallsworth, 1998; Hallsworth *et al*., 2007) when the medium contained high concentrations of a compatible solute, proline (Thomas *et al*., 1993); furthermore of the seven microbes reported to have the highest resistance to other chaotropic substances (including urea, phenol) the majority are weed species including *A. wentii, S. cerevisiae, Escherichia coli* and *P. putida* (Hallsworth *et al*., 2007).

The stress mechanisms for numerous types of cellular stress operate at the level of water:macromolecule interactions (Crowe, *et al*., 1984; Brown, 1990; Sikkema *et al*., 1995; Casadei *et al*., 2002; Ferrer *et al*., 2003; Hallsworth *et al*., 2003a; 2007; McCammick *et al*., 2009; Bhaganna *et al*., 2010; Chin *et al*., 2010). As front-line stress protectants, compatible solutes (within limits) can therefore mollify stress the inhibitory effects of an indefinite number of stress parameters (see Crowe, *et al*., 1984; Brown, 1990; Hallsworth *et al.,* 2003b; Bhaganna *et al*., 2010; Chin *et al*., 2010; Bell *et al*., 2013) but cells also upregulate other protection systems (see also above; Table 3). In *S. cerevisiae*, an extensive number of genes that code for protein-stabilizing proteins, metabolic enzymes and cellular-organization proteins are regulated by a single promoter – the stress response element (STRE) – which is activated under cellular stress. This regulatory system enables the coordination of an array of stress responses in an energy-efficient manner (Table 3). Numerous studies show that the *S. cerevisiae* responses to osmotic stress [via the high-osmolarity glycerol-response (HOG) pathway], oxidative stress, and challenges to protein and membrane stability are exceptionally efficient (Table 3; Brown, 1990; Costa *et al*., 1997; Hallsworth, 1998; Rodríguez-Peña *et al*., 2010; Szopinska and Morsomme, 2010). In addition to these stress responses, there is evidence that the dynamics of polyphosphate and glycogen metabolism in *S. cerevisiae* are wired in such a way to boost ATP production (Castrol *et al*., 1999), and that the ploidy levels of many strains enhance tolerance towards oxidative and other stresses (Table 3). The energy-generation and energy-conservation strategies employed by *S. cerevisiae* have apparent parallels in other weed species; for example, analyses of synonymous codon bias in the genomes of fast-growing bacteria including *E. coli* and *Bacillus subtilis* suggest that genes associated with energy generation are highly expressed (Karlin *et al*., 2001; Table 5; see also above). The enhanced energy efficiency that is apparent in some weed species not only underpins their robust stress biology, but is required to sustain a vigorous growth phenotype and/or to out-grow competitors.

**Table 5:** Growth, nutritional versatility and resource acquisition and storage: cellular characteristics that contribute to weed-like behaviour.^a^

Metabolites, proteins, molecular characteristics, and other traits	Function(s)	Phenotypic traits	Notes and references
**Ability to out-grow competitors**			
Multiple copies of ribosomal DNA operons	Enhanced production of ribosomes	Rapid protein synthesis	Soil bacteria able to grow rapidly on high-nutrient media have an average of ∼ 5.5 copies of ribosomal DNA operons (compared with ∼ 1.4 copies for slow-growing species; Klappenbach *et al*., 2000)
Key roles of di-/tripeptide-transport protein (*dtp*T) and oligopeptide transport ATP-binding protein (Opp)	Permease (*dtp*T) and ABC-type transporter (Opp) that enable efficient scavenging of diverse peptides (Altermann *et al*., 2005)	Efficient uptake of diverse peptides	These proteins enable species such as *Lactobacillus acidophilus* to efficiently scavenge peptides and thereby minimize the need for *de novo* synthesis of amino acids (Altermann *et al*., 2005)
High-efficiency DNA polymerase	Apparent correlation with short generation-time in fast-growing bacteria (Couturier and Rocha, 2006)	Rapid genome replication	The relatively fast-growing *Mycobacterium smegmatis* (generation time ∼ 3 h) has a DNA polymerase processing rate of 600 nucleotide s^−1^, compared with 50 nucleotide s^−1^ for *Mycobacterium tuberculosis* (generation time ∼ 24 h; Straus and Wu, 1980; Couturier and Rocha, 2006)
Overlapping replication cycles	Bacteria can initiate a new round of chromosomal DNA replication before the completion of an ongoing round of synthesis	Rapid genome replication	*Escherichia coli* can operate up to eight origins of replication simultaneously (see Nordström and Dasgupta, 2006)
Polyploidy, aneuploidy, association with plasmids, and/or hybridization that enhance(s) vigour	Multiple copies of genomic DNA can lead to increased vitality, enhanced growth rates, increased production of antimicrobials, and enhanced energy generation and stress tolerance (see also Table3)	Enhanced vigour	Shorter generation times in polyploid bacteria may arise from a number of factors including the increased number of ribosomal DNA operons (Cox, 2004; see also above) and gene localization within the genome (Pecoraro *et al*., 2011). A recent study of F1 hybrids produced by crossing 16 wild-type strains of *S. cerevisiae* showed that many F1 strains displayed hybrid vigour (Timberlake *et al*., 2011)^b^>
Traits for high-efficiency energy generation (see Table3)			
Efficient system to eliminate free radicals (see also Table3)	Elimination of reactive oxygen species that can react with and damage macromolecular and cellular structures	Ability to cope with the oxidative stress associated with high growth rates	The enhanced rates of oxidative phosphorylation required for rapid growth result in the increased generation of free radicals; a phenomenon that has been observed in plant weeds (Wu *et al*., 2011). *Aspergillus* mutants that lack superoxide dismutase genes are unable to grow under environmental conditions that favour a high metabolic rate (Lambou *et al*., 2010). Strains of *Pseudomonas aeruginosa* have an auxiliary manganese-dependent version of superoxide dismutase that does not require iron and is upregulated under iron-limiting conditions (Britigan *et al*., 2001)
**Nutritional and metabolic versatility**^c^			
Genes for catabolism of a wide spectrum of hydrocarbons; e.g. those coding for the catechol-, homogentisate-, phenylacetate- and protocatechuate-catabolic pathways (*cat*, *hmg*, *pha* and *pca* gene families respectively)	Facilitate the synthesis of proteins that enable the utilization of hydrocarbons (including polycyclic aromatic hydrocarbons)	Ability to utilize diverse hydrocarbons as a carbon and/or energy source	Such compounds can be utilized as a sole carbon source by a small number of metabolically versatile microbes such as *Pantoea* spp., *Pseudomonas putida* and *Pichia anomala* (Timmis, 2002; Pan *et al*., 2004; Vasileva-Tonkova and Gesheva, 2007) but are also known to exert cellular stress even at low concentrations (Bhaganna *et al*., 2010). Catabolism of hydrocarbons will therefore alleviate stress at the same time as providing for energy generation and growth (Jiménez *et al*., 2002; Domínguez-Cuevas *et al*., 2006)
Catabolite-repression system for energy-efficient utilization of complex substrates	Advanced system to regulate utilization of diverse substrates	Energy-efficient growth in nutritionally complex environments	A cAMP-independent catabolite-repression system has been identified in *P. putida* that enables the ordered assimilation of carbon, nitrogen and sulfur substrates (most energy-favourable first; Daniels *et al*., 2010)
Production and secretion of biosurfactants (e.g. rhamnolipids in *Pantoea*, *P. aeruginosa* and *P. putida*; glycolipoproteins in *Aspergillus* spp*.*)	Solubilization of hydrophobic molecules (and antimicrobial activity; see Table6)	Ability to enhance the bioavailability of hydrophobic substrates	*In vitro* studies of *Pantoea* and *P. aeruginosa* demonstrate that biosurfactants enhance the uptake of hydrophobic substrates (Al-Tahhan *et al*., 2000; Vasileva-Tonkova and Gesheva, 2007; Reis *et al*., 2011) and thereby facilitate colonization of hydrocarbon-containing habitats (see Table S1). *Aspergillus* species can produce copious amounts of biosurfactant (Desai and Banat, 1997; Kiran *et al*., 2009)
Diverse saprotrophic enzymes (e.g. cellulose, ligninase) able to function over a range of conditions	Extracellular degradation of polymeric organic molecules for use as a carbon and energy source	Ability to grow saprotrophically	Saprotrophic capabilities enhance the range of substrates from which nutrition can be obtained. Saprotrophic enzymes from *Aspergillus* species can function over a wide range of environmental stresses (Hong *et al*., 2001)
Proteins for oxidation of methane and/or methanol (e.g. alcohol oxidase in *Pichia* and *Kodamaea*)	Oxidation of one-carbon substrates that are used for energy generation/growth	Ability to utilize one-carbon substrates	One-carbon substrates are environmentally commonplace and their utilization can facilitate rapid growth (van der Klei *et al*., 1991; McDonald and Murrell, 1997; Pacheco *et al*., 2003)
Pathways to utilize a broad range of nitrogen substrates (e.g. D-amino acids, urea, ammonium salts, and macromolecules such as collagen and elastin)	Assimilation of nitrogen into anabolic pathways for *de novo* synthesis of amino acids and nucleic acids	Ability to utilize a wide spectrum of nitrogen sources	Versatility in the utilization of nitrogen substrates can enable proliferation in open habitats by, and opportunistic pathogenicity for, *Aspergillus*, *Pseudomonas putida* and other weed species (Krappmann and Braus, 2005; Daniels *et al*., 2010; see also Table S1). *Rhodotorula* spp., including metabolically versatile weed species, can grow on D-amino acids as a sole carbon and nitrogen source (Simonetta *et al*., 1989; Pollegioni *et al*., 2007)
Genome-mediated responses to maintain growth under low-nutrient conditions (e.g. upregulation of glutamate dehydrogenase in *P. putida*)	Glutamate dehydrogenase plays key roles in nitrogen assimilation and metabolism	Ability to adapt metabolism for growth at high- or low-nutrient concentrations	Expression of *gdh* was between 5- and 26-fold greater in *P. putida* cells under low-nutrient conditions compared with a high-nutrient control (Syn *et al*., 2004). The synthesis of glutamate via glutamate dehydrogenase, rather than glutamate synthase, consumes 20% less ATP (Syn *et al*., 2004)
Enzyme systems that enable metabolism under nutrient-limiting conditions by avoiding dependence on specific cofactors	Utilization of substitute cofactor, or replacement of enzyme system(s) that would require a cofactor that is environmentally scarce	Maintenance of essential enzyme functions at low nutrient concentrations	For example arginase in *S. cerevisiae* (Middelhoven *et al*., 1969) and phospholipase C in *P. aeruginosa* (Stinson and Hayden, 1979)
Pathways to synthesize phosphate-free membrane lipids	Maintenance of membrane functions under phosphate-limited conditions	Ability to remain active, and grow, in phosphate-depleted environments	*Pseudomonas* spp. can synthesize membranous glycolipids as an alternative to phospholipids under low-phosphate conditions (Minnikin *et al*., 1974). *Salinibacter ruber* and a number of phytoplankton species utilize sulfur in membrane glycolipids that have a sulfoquinovose-containing (non-phosphorus) head group and are synthesized in response to low phosphate availability (Benning, 1998; Corcelli *et al*., 2004; Bellinger and Van Mooy, 2012)
High glucosylglycerate under low-nitrogen conditions	Control of nitrogen homeostasis to enable growth under nitrogen-limiting conditions	Nitrogen oligotrophy	Intracellular glucosylglycerate is increased in *M. smegmatis* under low nitrogen conditions. Mutants unable to synthesize glucosylglycerate showed reduced uptake of ammonium and a reduced growth rate (Behrends *et al*., 2012)
**Efficient acquisition and storage of resources**			
Transport proteins to scavenge peptides and/or other macromolecules	Efficient scavenging of biomacromolecules avoiding the need for *de novo* synthesis	Efficient uptake of substrates	For example in *L. acidophilus* (see also above; Altermann *et al*., 2005)
Biosurfactants to access hydrophobic substrates (see above)			
Production of high quantities of high- affinity saproptrophic enzymes	Efficient degradation of complex extracellular substances	Efficient utilization of high-*M*_r_ substrates	For example. xylanases produced by *Acinetobacter junii* and *Aspergillus* spp. (Meagher *et al*., 1988; Lo *et al*., 2010)
High-affinity hexose-transport proteins, e.g. Hxt2p (*HXT* gene family)	Highly efficient uptake of diverse carbon substrates	Rapid uptake of hexose sugars	These proteins are upregulated in *S. cerevisiae* at low sugar concentrations (Wendell and Bisson, 1994)
Phytase, inorganic phosphate transport proteins and polyphosphate kinase	Extracellular degradation of organic phosphate and uptake and intracellular polymerization of inorganic phosphate	Sequestration and storage of inorganic phosphate	*Pichia kudriavzevii* has a high rate of inorganic phosphate production from phytate (Hellström *et al*., 2012). Reduced survival of polyphosphate kinase-deficient bacterial mutants has been observed at low pH, high salinity, and under oxidative stress (Jahid *et al*., 2006). Other weed species, such as *P. putida, Dunaliella salina*, *Microcystis aeruginosa* and *Ostreococcus tauri* are efficient accumulators of polyphosphate (Bental *et al*., 1991; Shi *et al*., 2003; Tobin *et al*., 1997; 2007; Le Bihan *et al*., 2011)
Production and secretion of siderophores that chelate iron	To scavenge iron from the extracellular milieu which is then removed once the siderophores have been transported back into the cell	Efficient scavenging of iron	The competitive ability of some bacterial weeds (e.g. pseudomonads, *Pantoea ananatis*) may be enhanced by siderophore production (Duiff *et al*., 1993; Loaces *et al*., 2011). *Rhodotorula mucilaginosa* and *M. aeruginosa* upregulate siderophore production under iron-limiting conditions (Andersen *et al*., 2003; Xing *et al*., 2007); in contrast, *Saccharomyces* spp. are not known to produce siderophores (Lesuisse *et al*., 2001)
Enhanced efficiency of iron-binding proteins (e.g. transferrin-like protein, TTf, in *D. salina*)	Binding and uptake of extracellular iron	Ability to accumulate iron under iron-limiting conditions	*Dunaliella salina* cells undergo a fourfold increase in iron-binding affinity under low-iron conditions. Iron is then taken up stored in vacuoles (Paz *et al*., 2007)
Arginine biosynthesis proteins	Bulk synthesis of arginine as a nitrogen substrate store	Amino acid accumulation and storage	*Saccharomyces cerevisiae* stores arginine (in vacuoles) that can be utilized when the extracellular environment becomes nitrogen substrate limited (Westenberg *et al*., 1989)
Trehalose-synthesis proteins (e.g. TPS, TPP) and glycogen synthase (e.g. GSY1 and GSY2)	Synthesis of disaccharide- and polysaccharide-storage compounds (e.g. trehalose and glycogen respectively)	Effective synthesis and storage of carbohydrates	Trehalose synthesis can be upregulated prior to the stationary growth phase and during stress (Parrou *et al*., 1997), and glycogen synthesis is upregulated during periods of nutrient limitation, in *S. cerevisiae* (Lillie and Pringle, 1980; Farkas *et al*., 1990). Starch is accumulated by *D. salina* (Stoynova-Bakalova and Toncheva-Panova, 2003)
Polyhydroxybutyrate-synthesis proteins (and those producing other storage lipids)	Synthesis of lipid stores (see also Table3)	Bulk storage of lipids	Polyhydroxybutyrate, in the form of intracellular granules, dominates the cytoplasm of *Haloquadratum walsbyi* (Saponetti *et al*., 2011); *Dunaliella* spp. also store lipids (Chen *et al*., 2011); recent studies of *P. putida* suggest that carbon and energy can be channelled into storage of polyhydroxybutyrate without a reduction of growth rate (Escapa *et al*., 2012)
Morphological and physiological adaptations to optimize light capture and absorption of oxygen and nutrients	Flat cell shape gives a high surface-to-volume ratio; gas vesicles help to maintain the horizontal orientation of planktonic cells	Optimized light capture, and oxygen and nutrient absorption	The remarkable morphology of *H. walsbyi* cells optimizes acquisition of resources in highly competitive high-salt habitats (Bolhuis *et al*., 2006; Bardavid *et al*., 2008)
Efficient chlorophyll *a* and chloroplast arrangement	Optimization of light harvesting and high photosynthetic rate	High photosynthetic rate	Habitat dominance by *Gonyostomum semen* can be attributed, in part, to having a higher photosynthetic rate than other autotrophic species (Coleman and Heywood, 1981; Peltomaa and Ojala, 2010)
Genes related to gas synthesis and gas vesicle formation (e.g. *gvp* family in *M. aeruginosa*)	Gas vesicles provide buoyancy to optimize access to light and high oxygen concentrations (close to the water:atmosphere interface of aquatic habitats)	Ability to access resources required for photosynthesis	Twelve genes involved in gas synthesis and storage have been identified in *M. aeruginosa* (Mlouka *et al*., 2004); models suggest regulation of buoyancy in *M. aeruginosa* has a key role in habitat dominance (Bonnet and Poulin, 2002)

a The current table provides examples of proteins, genes or other characteristics that can be associated with and give rise to weediness; the categories of traits are not mutually exclusive. Individual traits may not be unique to microbial weeds; however, weeds species are likely to have a number of these types of characteristics.

b Whereas polyploidy can enhance vigour (via increased growth rates, enhanced stress tolerance etc.) increased chromosome number can also result in decreased growth rates; the consequences of polyploidy can vary with species and environmental context.

c Nutritional versatility is particularly advantageous for microbial weed species that have a generalist lifestyle such as *Pseudomonas putida* and *Pichia anomala* (Timmis, 2002; Walker, 2011).

### Growth, nutritional versatility, and resource acquisition and storage

Efficient cell division and an ability to out-grow competitors are implicit to the emergence of weed species. Species that grow relatively slowly or remain quiescent, and those with specific requirements for a host, a symbiotic partner, or obligate interactions with other microbial species are disadvantaged during community development in open habitats: dominance ultimately results from vigour and – in many habitats – a degree of nutritional versatility (Tables S1 and5). Ability to out-grow competitors is equally important (possibly even more so) for weed species in nutrient-poor and physicochemically extreme habitats that can both select for slow-growing species and limit overall biomass development. Many traits that give rise to an efficient growth phenotype can be found in weed species (Table 5): multiple copies of ribosomal DNA operons that can enhance rates of protein synthesis; transport proteins to scavenge macromolecules thus negating the need for *de novo* synthesis; high-efficiency DNA polymerase for rapid genome replication; efficient systems to eliminate reactive oxygen species that are a by-product of rapid growth; and polyploidy, aneuploidy and plasmids that can be associated with enhanced stress tolerance, higher growth-rates and increased overall vigour (Table 5). We believe that another key aspect of weed biology for some microbial species is their ability to sequester and store available nutrient and substrate molecules with a high efficiency as an auxiliary energy reserve (see below), to be able to supply daughter cells with nutrients, and simultaneously deprive other microbes of nutritional resources (e.g. Duiff *et al*., 1993; Castrol *et al*., 1999; Loaces *et al*., 2011; Renshaw *et al*., 2002; Jahid *et al*., 2006). Microbial weeds are highly efficient at nutrient-sequestration and substrate utilization, as well as intracellular storage of these substances (Table 5). For example, fungal siderophores that remove iron from the habitat have been shown to suppress growth of other microorganisms due to iron limitation (Renshaw *et al.,* 2002).

Intermittent nutrient availability and low nutrient concentrations are characteristic of many aquatic environments so the ability to scavenge and store, circumvent the requirement for, or strategically utilize scarce and potentially limiting nutrients can greatly enhance the competitive ability of planktonic species. Under phosphate-limiting conditions some aquatic species, including *S. ruber*, are able to use sulfur-containing glycolipids in place of phospholipids during low phosphate availability (Table 5). The cyanobacterium *M. aeruginosa* has high-efficiency phosphate-transport systems and accumulates intracellular polyphosphate which is used to maintain activity once extracellular phosphate is depleted (Table 5; Shi *et al*., 2003). It is likely that this trait contributes to its weed-like phenotype in Lake Taihu (China) that is a well-studied, phosphate-limited habitat frequently dominated by *M. aeruginosa* (Shi *et al*., 2003). Iron is another potentially limiting nutrient in aquatic habitats. *Dunaliella salina* utilizes a highly efficient iron-binding protein to take up iron which is then stored in vacuoles (Paz *et al*., 2007). Species such as *D. salina* and *H. walsbyi* cope with oligotrophic conditions by hyperaccumulating starch and/or lipids such as polyhydroxybutyrate especially when nutrients other than carbon are limiting (Table 5; Bardavid *et al*., 2008). One weed species with adaptations to endure low-nitrogen conditions is *Mycobacterium smegmatis*. This species (as well as other mycobacteria) which can be prevalent in relatively low-nutrient aquatic and non-aquatic habitats (Koch, 2001; Kazda and Falkinham III, 2009) have biochemical adaptations to enable growth and metabolic activity even when nitrogen concentrations are an order of magnitude lower than those usually required for structural growth (i.e. a C : N ratio of 8:1; Roels, 1983; Behrends *et al*., 2012). In addition to nitrogen oligotrophy*, M. smegmatis* has a DNA polymerase capable of nucleotide processing rates and generation times that are an order of magnitude faster than those of comparable *Mycobacterium* species (Table 5). Light and/or oxygen are key resources for many planktonic species; 12 genes have been identified that are involved in gas synthesis and storage in species such as *M. aeruginosa* and *H. walsbyi* (Table 5). Modelling approaches indicate that buoyancy, which enables the cell to access the upper (well-illuminated, well-oxygenated) section of the water column, plays a key role in habitat dominance by *M. aeruginosa* (Bonnet and Poulin, 2002). Studies of *H. walsbyi* suggest that its peculiar, flat cell-shape optimizes the uptake of nutrients and oxygen and its intracellular gas vesicles help to maintain a horizontal orientation to capture light (Table 5). In freshwater habitats dominance of algal blooms by *G. semen* has been attributed in part to a high photosynthetic rate that results from a combination of its high-efficiency chlorophyll *a* and a highly efficient chloroplast arrangement (Table 5).

In open habitats that are nutritionally dynamic and/or complex (Table S1) metabolic versatility in nutrient utilization can enhance the competitive ability of microbes (for examples see Table 5). *Pseudomonas putida* and related species are remarkable in their range of nutritional strategies (Table 5; Timmis, 2002; 2009): they are able to catabolize an indeterminate number of hydrocarbons (e.g. decanoate, toluene) and other organic molecules (e.g. amino acids, and organic acids such as acetic, citric and lactic acid) for use as carbon and energy sources; amino acids, dipeptides, ammonium chloride, urea, ammonium nitrate, and other substances as nitrogen sources; amino acids, organic acids, sodium sulfite, thiouracil, agmatine sulfate, etc. as sulfur sources; as well as accumulating diverse substances as intracellular reserves (Chakrabarty and Roy, 1964; Daniels *et al*., 2010; Table 5) enabling a switch to oligotrophic growth at low nutrient concentration (Table 5; Timmis, 2002). In addition, *P. putida* and closely related species have evolved a complex, cAMP-independent catabolite repression system which incorporates five different signals and enables an ordered assimilation of carbon, nitrogen and sulfur substrates from the most to least energetically favourable (Daniels *et al*., 2010). This may contribute to the ability of many pseudomonads to sustain vigorous growth in nutritionally complex habitats. Pseudomonads, and closely related bacteria such as *Acinetobacter,* are not only environmentally ubiquitous but can dominate microbial communities in numerous types of open habitat including the plant phyllosphere and rhizosphere, bioaerosols, oil sands, and other hydrocarbon-containing environments (see Table S1; Ogino *et al*., 2001; Röling *et al*., 2004; Louvel *et al*., 2011). *Pseudomonas* species are able to enhance the availability of hydrophobic substrates by secreting biosurfactants and, at the same time, tolerate the stress induced by the latter (see below). Despite their copiotrophic phenotype, pseudomonads utilize a number of strategies to maintain growth and metabolism under phosphate-, iron- or nitrogen-depleted conditions (Table 5), can synthesize glutamate via glutamate dehydrogenase rather than glutamate synthase (thereby consuming 20% less ATP; Syn *et al*., 2004), and have even been observed growing in distilled water (Favero *et al*., 1971; Hirsch, 1986). Specific enzymes of *Pseudomonas aeruginosa* are catalytically active even when cofactor nutrients are scarce, and some strains have a manganese-dependent superoxide dismutase which does not require iron and is upregulated at low iron concentrations (Table 5).

Weeds such as lactic acid bacteria and *S. cerevisiae* that lack saprotrophic capabilities can dominate communities in habitats with high nutrient concentrations (Tables S1 and5); they possess high-affinity transport proteins that enable rapid and efficient uptake of substrate molecules to support their vigorous growth phenotypes (see Table 5). Cells of *S. cerevisiae* are not only highly efficient at storing carbohydrates (combined trehalose and glycogen may reach 25% dry weight; Aranda *et al*., 2004; Guillou *et al*., 2004), but they can also accumulate high concentrations of arginine and polyphosphate and have adaptations to retain activity under low-nitrogen conditions (Table 5). Plant weeds that excel in open habitats have distinct genetic properties; they are often of hybrid origin and/or polyploid, traits that give rise to increased growth rates and stress tolerance, and have specific genes that confer their vigorous phenotype (Anderson, 1952; Hill, 1977; Schnitzler and Bailey, 2008; Bakker *et al*., 2009). There is also evidence that the multiple copies of genomic material found in vigorous microbes that enhanced vigour have arisen via hybridization and/or increased ploidy levels in bacterial weed species, as well as *Pichia* and *Saccharomyces* (Table 5; Cox, 2004). A recent study by Timberlake and colleagues (2011) reported vigorous growth for the majority of F1 hybrids produced by crossing 16 wild-type *S. cerevisiae* strains (although hybridization and/or polyploidy may not always be advantageous; see Table 5). Many microbial weed species are phenotypically variable (like their plant counterparts; Table 1; Hill, 1977) and may form part of a species complex due to polyploidization and hybridization (e.g. *Acinetobacter, Pseudomonas, Mycobacterium, Saccharomyces sensu stricto*; Table S1; Rainieri *et al*., 1999; Todd *et al*., 2004; Ballerstedt *et al*., 2007; Parkinson *et al*., 2011; Sicard and Legras, 2011); such factors may contribute to the vigour and weediness of such species.

High-*M*_r_ macromolecules are the primary microbial substrates available in many habitats: e.g. hydrocarbons (in bioaerosols, oil-containing environments, and the rhizosphere), and polysaccharides (within or derived from plant necromass such as cellulose, lignin and humic substances; see Table S1). In order to out-compete other species in such habitats, microbes must utilize these macromolecular substrates that typically have a low bioavailability, require specialist enzymes to degrade, can be toxic, and/or are energy-expensive to catabolize. Facultatively saprotrophic microbes such as *Aspergillus, Pichia, Rhodotorula* and *Acinetobacter* species are able to access and catabolize recalcitrant polysaccharides; some weed species can produce exceptional amounts, or high-affinity versions, of inducible saprotrophic enzymes, such as xylanases (Table 5). Several saprotrophic enzymes produced by *Aspergillus* species (that can dominate physicochemically diverse habitats; Table S1) can retain activity over a wide range of environmental stresses (Hong *et al*., 2001); and *Aspergillus,* and *Rhodotorula* spp. are able to utilize a broad range of nitrogen substrates thereby avoiding the need for *de novo* synthesis (Table 5). Whereas nutritionally complex habitats can select for metabolic versatility (see below) and thereby favour the growth of a great number of generalist species, only a minute fraction of such microbes are able to dominate what were (initially) phylogenetically diverse microbial communities (Table S1).

### Antimicrobial strategies and trophic interactions

Grazing, predation and viral infections adversely impact the growth dynamics of and/or incur massive losses on microbial populations and so exert a powerful selective pressure, and this can be well illustrated by the ecologies of many planktonic species in aquatic habitats (Matz *et al*., 2004; Jüttner *et al*., 2010; Berdjeb *et al*., 2011; Thomas *et al*., 2011; Yang and Kong, 2012). The competitive ability of some aquatic bacteria and eukaryotes (both marine and freshwater species) is associated with their ability to resist or tolerate virus infection (see Table 6). Species with a halophilic growth phenotype, especially those which have high growth rates in salt-saturated environments such as *S. ruber* and *H. walsbyi* minimize losses to predators because these salt concentrations are hostile to protozoa due to their low water activity, high ionic strength, and/or (for high-MgCl_2_ and high-CaCl_2_ brines) high chaotropicity (Table 6; Hallsworth *et al*., 2007). *Gonyostomum semen* has evolved vertical migration patterns through the water column that are out of phase with those of grazing species and thereby minimizes losses (Table 6). Other weeds, including *P. aeruginosa* and *M. aeruginosa*, are capable of forming multicellular structures – micrcolonies – that are sufficiently large to reduce losses by many species of grazers and predators (Table 6). Species such as *M. aeruginosa* accumulate toxic metabolites, and *M. aeruginosa* and *Aspergillus* spp. are known to synthesize secondary metabolites that repel to grazing plankton and arthropods respectively (Table 6).

**Table 6:** Antimicrobial activities and trophic interactions: cellular characteristics that contribute to weed-like behaviour.^a^

Metabolites, proteins, molecular characteristics, and other traits	Function(s)	Phenotypic traits	Notes and references
**Avoidance of/resistance to viral infection, predators, and grazers**			
Metabolites that repel grazers and/or predators, e.g. aldehydes and other volatile organic compounds in plankton and fungi	Secondary metabolites produced by *Aspergillus* spp. repel (and can reduce the fecundity of) grazing arthropods such as *Folsomia candida*; those produced by *Microcystis aeruginosa* act as a repellent to planktonic grazers	Ability to repel grazers and predators	*Aspergillus nidulans* mutants lacking the *laeA* gene, a global regulator of secondary metabolite synthesis, are consumed by *F. candida* in preference to the wild-type strain (Rohlfs *et al*., 2007). Aldehydes produced by plankton repel crustacean grazers (Jüttner, 2005); β-cyclocitral from *M. aeruginosa* has been shown to repel *Daphnia magna* (Jüttner *et al*., 2010)
Synthesis of extracellular polymeric substances (e.g. *M. aeruginosa*), or use of flagella (e.g. *Pseudomonas aeruginosa*), that facilitate microcolony formation	Formation of multicellular structures that are sufficiently large to minimize grazing and predation by single-celled protozoa, zooplankton etc.	Microcolony formation	The role of polymeric substances in colony formation has been established using *M. aeruginosa* as a model organism (Yang and Kong, 2012). Functional analysis of *P. aeruginosa* mutants that were unable to form flagella, or were alginate-deficient, demonstrated an inability to form microcolonies that can confer resistance to grazing (Matz *et al*., 2004)
Biochemical adaptations to resist viral infection	Diverse mechanisms including modification of cell surface to prevent binding, intracellular suppression or viral genome replication, and degradation of viral DNA by restriction endonucleases	Resistance to or tolerance of viral infection	There are a number of mechanisms by which dominating species of bacteria and eukaryotic plankton (e.g. *P. aeruginosa, Ostreococcus tauri; Bathycoccus* and *Micromonas* spp.) may acquire and maintain resistance to viral infection (see Thomas *et al*., 2011; Selezska *et al*., 2012)
Toxic metabolites (e.g. microcystin in *M. aeruginosa*)	Toxicity against grazing or predatory zooplankton	Ability to protect against excessive grazing	Microcystin synthesis in *M. aeruginosa* can be induced by exposure to zooplankton; concentrations of the toxin in cells exposed to *D. magna*, *Daphnia pulex* and *Moina macrocopa* were up to fivefold those found in control cells (Jang *et al*., 2003)
Halophilic growth phenotype	Colonization of salt-saturated habitats that are hostile to predatory protozoa minimizes losses from grazing and predation	Ability to inhabit environments hostile to predators and grazers	Halophiles that dominate high-salt habitats (e.g. *D. salina*, *S. ruber*) minimize interactions with grazers and predators most of which cannot tolerate molar salt concentrations (Bardavid *et al.,* 2008)
Biochemical and cellular infrastructure that give rise to idiosyncratic migration patterns in the water column	Vertical migration within the water column that minimizes loss through predation and grazing	Avoidance of predators and grazers	The predominance of *Gonyostomum semen* in eutrophic habitats has been associated in part with migration patterns that minimize loss through grazing (Salonen and Rosenberg, 2000)
**Bulk production of highly soluble, moderately chaotropic stressors**^b^			
Ethanol produced via a high-K_m_ alcohol dehydrogenase (e.g. Adh1 in *Saccharomyces cerevisiae*)	Efficient conversion of acetaldehyde to ethanol; in contrast, low-K_m_ Adh typically converts ethanol to acetaldehyde under *in vivo* conditions (Piškur *et al*., 2006)	Production and secretion of ethanol (up to molar concentrations)	Ethanol, secreted in quantity by species such as *S. cerevisiae* and *Zymomonas mobilis*, is a moderately chaotropic substance that is nevertheless a potent stressor at millimolar to molar concentrations (Hallsworth, 1998; Bhaganna *et al*., 2010; Cray *et al*., 2013)
Enzymes for acetone, isopropanol and butanol synthesis	Synthesis of chaotropic substances in the anaerobic bacterium *Clostridium acetobutylicum*	Bulk production, and secretion, of multiple chaotropic stressors	Acetone, isopropanol and butanol that are produced by *C. acetobutylicum* (George *et al*., 1983) are more chaotropic ethanol – when compared on a molar basis – and are therefore more potent in their inhibitory activity as cellular stressors (Bhaganna *et al*., 2010; Cray *et al*., 2013; Bell *et al*., 2013)
Production of high concentrations of urea and ammonia	Synthesis of low-*M*_r_, nitrogenous chaotropes	Production and secretion of urea and ammonia at inhibitory concentrations	Urea and ammonia are produced by phylogenetically diverse bacteria such as *Pseudomonas stutzeri* and *Clostridium aminophilum* respectively (Therkildsen *et al*., 1997; Watanabe *et al*., 1998; Anderson *et al*., 2010)
**Release of VOCs as low-solubility antimicrobial stressors**			
Highly volatile metabolites that act as hydrophobic stressors (log P > 1.95)^c^	Inhibition of microbial metabolism; typically via hydrocarbon-induced water stress (Bhaganna *et al*., 2010)	Production and release of volatile antimicrobials	Examples are: isoamyl acetate (produced by *Pichia anomala*; Passoth *et al*., 2006), ethyl octanoate (*S. cerevisiae*; Fialho *et al*., 2011), β-cyclocitral (*M. aeruginosa* and *Dunaliella salina*; Herrero *et al*., 2006; Ozaki *et al*., 2008), pentane (*D. salina*; Muñoz *et al*., 2004); and diverse substances from *Aspergillus* (see above), *R. mucilaginosa* (Buzzini *et al*., 2005), *P. putida* and other *Pseudomonas* species (Morales *et al*., 2005)
Highly volatile, strongly chaotropic metabolites (log P typically in the range 1–1.9)^d^	Inhibition of microbial metabolism via chaotrope-induced water stress (Hallsworth, 1998; Hallsworth *et al*., 2003a; Bhaganna *et al*., 2010)	Production and release of volatile antimicrobials	Examples are: ethyl acetate (produced by *Pichia anomala*; Walker, 2011), dichloromethane (*Dunalialla* spp.; Colomb *et al*., 2008) and 2-phenylethanol (*Mycobacterium bovis, Mycobacterium smegmatis* and *S. cerevisiae;* Eshkol *et al*., 2009; McNerney *et al*., 2012); and a range of chaotropic stressors by *R. mucilaginosa* (Buzzini *et al*., 2005), *P. putida* and other *Pseudomonas* species (Morales *et al.,* 2005)
Volatile catabolites produced from the degradation of macromolecular substrates	Inhibition of microbial metabolism typically via chaotrope-induced or hydrocarbon-induced water stress (Hallsworth *et al*., 2003a; Bhaganna *et al*., 2010)	Production and release of volatile antimicrobials^g^	Antimicrobial stressors can be released during the saprotrophic degradation of lignin, catabolism of hydrocarbons etc. (e.g. catechol, vanillin and biphenyl in *P. putida*; Zuroff and Curtis, 2012)
**Acidification of the extracellular environment**			
Secretion of organic acids	Acidification of the extracellular milieu	Ability to acidify the habitat	*Acinetobacter rhizosphaerae* and *S. cerevisiae* secrete a range of substances that can acidify the environment (Kotyk *et al*., 1999; Gulati *et al*., 2010); *Aspergillus* spp. produces high levels of citric acid (up to 200 g l^−1^ in 5–7 days; Ward *et al*., 2006)
High-activity l-lactate dehydrogenase (e.g. ldhL in *Lactobacillus plantarum*)	Intracellular conversion of pyruvate to lactate	Production and secretion of lactic acid	The specific activity of IdhL from a lactic acid produced by *L. plantarum* was found to be 2460 U mg^−1^; compared with 250 U mg^−1^ for *Escherichia coli* (Garvie, 1980). Habitat acidification by lactic acid inhibits the growth of many microbial competitors (Table S1; Guillot *et al*., 2003). *Lactobacillus* efficiently acidifies its environment even at low sugar concentrations (Klinke *et al*., 2009)
Enzymes for acetic and butyric acid synthesis (e.g. phosphotransacetylase and acetate kinase, and phosphate butyryltransferase and butyrate kinase respectively)	Conversion of acetyl-CoA to acetate; and butyryl-CoA to butyrate	Production/secretion of acetic acid and butyric acid	*Clostridium* spp. produce acetic, butyric and lactic acids; fermentations carried out in media with an initial pH of ∼ 6.5 resulted in a final pH of 4–4.5 (Wiesenborn *et al*., 1989; Boynton *et al*., 1996; Liu *et al*., 2011)
**Production of biosurfactants, enzymes, and/or toxins with antimicrobial activities**			
Biosurfactants (e.g. rhamnolipids in *Pseudomonas* spp.; glycolipoproteins in *Aspergillus* spp.)	Solubilization of hydrophobic substrates; inhibitory to the growth of many microbial competitors	Production of inhibitory biosurfactants	Lipopeptides from *Pseudomonas* sp. DF 41 were shown to inhibit *Sclerotinia sclerotiorum* (Berry *et al*., 2010); purified biosurfactants from *Pseudomonas fluorescens* inhibit growth of *Botrytis cinerea* and *Rhizoctonia solani* at low concentrations (25 μg ml^−1^) and can lyse zoospores of *Phytophthora capsici* (Kruijt *et al*., 2009). Studies of *P. aeruginosa* rhamnolipid suggests a key role in competitive ability against other bacteria (Wecke *et al*., 2011). *Acinetobacter* spp. produces a potent biosurfactant (phosphatidylethanolamine; Desai and Banat, 1997) which may act as an antimicrobial. Biosurfactants from *Aspergillus ustus* are inhibitory to phylogenetically diverse microbes (Kiran *et al*., 2009)
Panomycocin (an exo-β-1,3-glucanase)	Hydrolysis of glucans such as laminarin	Antimicrobial and cytocidal activity via degradation of cell wall components	Panomycocin is produced by *P. anomala* and is inhibitory or lethal to many species of yeasts and fungi such as *B. cinerea* (Izgü *et al*., 2005)
Ochratoxin and other mycotoxins (e.g. in *Aspergillus* spp.)	Inhibition of other microbial species/strains	Secretion of antimicrobials	Mycotoxins can inhibit phylogenetically diverse microbes (including fungal and bacterial species) and thereby enhance competitive ability (Boutibonnes *et al*., 1984; Magan *et al*., 2003)
Antifungal peptide (Anafp; in *Aspergillus niger*)	Mode-of-action not yet resolved	Secretion of an antifungal peptide	Anafp inhibits growth of yeasts and fungi including some *Aspergillus* and *Fusarium* spp., *Candida albicans, S. cerevisiae* and *Trichosporon beigelii* (Lee *et al*., 1999)
Mycocins (e.g. salt-mediated killer toxin)	Inhibitory to many fungi, yeasts and other microbes	Production of antibiotic peptides	A range of killer toxins produced by *Pichia farinose, Rhodotorula* spp. and *S. cerevisiae* that are inhibitory to phylogenetically diverse taxa (Golubev and Churkina, 1993;; Golubev *et al*., 1996; Suzuki *et al*., 2001; Trindade *et al*., 2002; Albergaria *et al*., 2010)
Bacteriocins e.g. plantarcins (*pln* gene family), lactacin-B (La1791–La1803), plant lectin-like bacteriocin (LlpA)	Modification of membrane structure causing leakage and dissipation of proton-motive force (Anderssen *et al*., 1998; Parret *et al*., 2003; Altermann *et al*., 2005)	Production of antibacterial peptides	For example *L. plantarum, Lactobacillus acidophilus* and *Pseudomonas* spp.
Pyoluteorin (in *P. putida*)	Inhibitory to growth of oomycetes and fungi; mode-of-action not yet resolved	Antifungal activity	Pyoluteorin-producing *P. putida* strains have potential as biocontrol agents of pathogens such as *Glomerella tucumanensis* (sugarcane red rot; Nowak-Thompson *et al*., 1999; Hassan *et al*., 2011)
Phenazine and phenazine-derived antimicrobials	Probable antimicrobial activity	Antimicrobial activity	Produced by species such as *M. aeruginosa* and *P. aeruginosa* (Mavrodi *et al*., 2006; Lifshits and Carmeli, 2012)
Mechanisms to induce cell lysis	Elimination of potential competitors	Algicidal activity	*Gonyostomum semen* is able to lyse microbial cells (e.g. *Rhodomonas lacustris*) on contact; mechanism not yet resolved (Rengefors *et al*., 2008)
**Resistance to antimicrobial stressors and toxins**			
Coordinated production of compatible solutes and ethanol	Protection against ethanol stress	Resistance to ethanol	The synthesis of glycerol (known to afford some protection against ethanol stress; Hallsworth, 1998; Hallsworth *et al*., 2003b) and ethanol production are coupled in *S*. *cerevisiae*; accumulation of trehalose (an effective protectant against ethanol; Mansure *et al*., 1994) coincides with the growth phase when ethanol concentrations are highest
Compatible-solute accumulation in response to solvent and hydrophobic stressors (see above)	Protection of macromolecular systems challenged by chaotropic and hydrophobic stressors (see studies of *A. nidulans*, *S. cerevisiae* and *P. putida*: Mansure *et al*., 1994; Hallsworth *et al*., 2003b; Park *et al*., 2007; Bhaganna *et al*., 2010; Bell *et al*., 2013)	Ability to maintain functionality of macromolecular systems	Kosmotropic compatible-solutes and other kosmotropic substances can reduce stress induced by chaotropic solutes such as MgCl_2_, glycerol^d^ and ethanol (Hallsworth, 1998; Hallsworth *et al*., 2007; Williams and Hallsworth, 2009). Tryptophan and trehalose are upregulated in cells of both eukaryotic and bacterial species in response to ethanol (Parrou *et al*., 1997; Purvis *et al*., 2005; Hirasawa *et al*., 2007; Horinouchi *et al*., 2010). *S. cerevisiae* could tolerate 28% v/v ethanol when protected by the kosmotropic compatible-solute proline (Thomas *et al*., 1993; Hallsworth, 1998)
Upregulation of protein-stabilization proteins (see Table3)	Protection of macromolecular systems from perturbations induced by antimicrobial stressors	Maintenance of protein structure and function during stressor-induced challenges	*Saccharomyces cerevisiae* upregulates synthesis of up to 10 heat-shock proteins under ethanol stress (Ma and Liu, 2010). *Pseudomonas putida* synthesizes diverse protein-stabilization proteins in response to chaotropic and hydrophobic stressors (see above; Hallsworth *et al*., 2003a; Bhaganna *et al*., 2010)
Membrane-stabilization proteins; solvent pumps (see Table3)			
Multidrug efflux pumps (e.g. AcrAB-TolC in *E. coli*; Pdr5p in *S. cerevisiae*; ABC transporter proteins in *Aspergillus* spp.)	Expulsion of chemically diverse stressors and toxins	Tolerance to a wide array of stressors and toxins	Including chaotropic solutes (e.g. phenol, benzyl alcohol), hydrophobic stressors (e.g. *n*-hexane, *p*-xylene) and antibiotics (e.g. chlorampenicol, tetracycline, novobiocin) by AcrAB-TolC; fluorescent dyes (e.g. daunorubicin, rhodamine), drugs (e.g. tamoxifen); and ionophoric peptides (e.g. nigericin) by Pdr5p (Kolaczkowski *et al*., 1996); and antifungals (cilofungin and echinocandin B) by the *AfuMDR1* gene product (Tobin *et al*., 1997; Nishino and Yamaguchi, 2001; Ramos *et al*., 2002)
Proteins for expulsion of, or resistance to, bacteriocins; e.g. lincomycin-resistance protein (lmrB), undercraprenyl pyrophosphate phosphatise (bacA)	Removal of bacteriocins from the cell	Resistance to bacteriocins	LmrB is an ATP-binding cassette-type transporter in *Lactococcus lactis* (Gajic *et al*., 2003); BacA is a membrane-associated protein that in *E. coli* for (El Ghachi *et al*., 2004)
Enzymes that inactivate antimicrobial substances	Detoxification and/or catabolism of inhibitory stressors and toxins	Ability to detoxify or degrade inhibitory antimicrobials	Examples include degradation of β-lactam antibiotics in *Acinetobacter* spp. and *P. aeruginosa* (Langaee *et al*., 2000; Sinha *et al*., 2007) and degradation of volatile stressors such as dichloromethane (e.g. *Bacillus* spp.; Wu *et al*., 2009)
Enhanced tolerance to a chaotropic volatile	Resistance to 2-phenylethanol	Resistance to VOCs	One strain of *S. cerevisiae* was found to be capable of growth at up to ∼ 24 mM 2-phenylethanol (Eshkol *et al*., 2009). For a chaotropic stressor, log P 1.52, almost as chaotropic as phenol, this is an exceptional level of tolerance (see Bhaganna *et al*., 2010)
Cell wall composition that confers resistance to specific antibiotics	Pathways targeted by specific antibiotics may be absent; cell wall can act as a barrier to antimicrobials	Resistance to antibiotics	The *Haloquadratum walsbyi* cell wall is not composed of peptidoglycan so this species has reduced sensitivity to β-lactam antibiotics (Bardavid and Oren, 2008); the *M. smegmatis* cell envelope contains mycolic acids that reduce permeability, and therefore enhance resistance, to stressors and antibiotics (Liu and Nikaido, 1999)
Synthesis of extracellular polymeric substances (see Table3)	Production and secretion of polymeric substances	Resistance to diverse antimicrobials	For example production of alginate by *P. aeruginosa* increases resistance to antibiotics such as tobramycin (Hentzer *et al*., 2001)

a The current table provides examples of proteins, genes or other characteristics that can be associated with and give rise to weediness; the categories of traits are not mutually exclusive. Individual traits may not be unique to microbial weeds; however, weeds species are likely to have a number of these types of characteristics.

b Chaotropic activity of > 5 and < 40 kJ kg^−1^ mole^−1^ (Cray *et al*., 2013).

c Volatile organic compounds are unlikely to exceed micromolar concentrations in the cytosol of the weed cell.

d Glycerol is a phylogenetically ubiquitous as a compatible solute for eukaryotic microbes, yet at molar concentrations it acts as a chaotropic stressor (Williams and Hallsworth, 2009; Cray *et al*., 2013).

A number of agricultural (plant) weeds are able to dominate their habitats due largely to the production of phytotoxic inhibitors that can be exuded from leaves, roots and plant necromass and which inhibit the germination and/or growth of other plant species (Hill, 1977; Xuan *et al*., 2006; Alsaadawi and Salih, 2009; Chou, 2010; Meksawat and Pornprom, 2010; Inderjit *et al*., 2011). It is likely that most microbial weed species also secure their competitive advantage – in part at least – via the deployment of antimicrobial substances. Assuming that the examples listed in Table S1 are representative of the microbial biosphere then the proportion of habitats dominated by eukaryotic species is approximately 40%. If a short generation time was sufficient to facilitate habitat domination then prokaryotic species would populate almost all these habitats. The fact that species of algae, fungi and yeast are also able to do so underscores the importance of factors other than generation time in the biology of habitat dominance. *Saccharomyces cerevisiae* provides an illustration of how one weed species combines the synthesis of a potent antimicrobial, energy generation, and coordinated synthesis of stress protectants in a single process, i.e. aerobic production of the chaotropic alcohol, ethanol (see above; Table 6). Ethanol can be produced and secreted at concentrations > 4 M that cause a reduction of water activity (to 0.896) and exert an exceptionally high chaotropic activity (see above) sufficient to prevent the growth or metabolism of any living system (Hallsworth, 1998; Hallsworth and Nomura, 1999; Hallsworth *et al*., 2007); *S. cerevisiae* simultaneously produces glycerol (which can reduce ethanol stress in yeast and fungi; Hallsworth, 1998; Hallsworth *et al*., 2003b) during ethanol fermentation and is metabolically wired to synthesize high concentrations of the protectant trehalose at the end of its growth cycle when ethanol concentration is highest (see Table 6).

We believe that the bulk production and secretion of chaotropic solutes and organic acids (that can reach molar concentrations) is unique to microbial weed species (Tables 1 and 2). Whereas chaotropic substances can promote growth and expand the biotic windows of microbes under specific conditions (Hallsworth and Magan, 1994b; 1995; Williams and Hallsworth, 2009; Bhaganna *et al*., 2010; Chin *et al*., 2010), in other circumstances chaotropicity can be highly inhibitory or even lethal (Hallsworth, 1998; Hallsworth *et al*., 2003a; 2007; Duda *et al*., 2004; Bhaganna *et al*., 2010). For plant weeds it is the early stages of habitat colonization that are key to their success (Hill, 1977), and is may be that the efficient synthesis of antimicrobials is frequently as important as much as the type of substance produced (Hallsworth, 1998; Pretorius, 2000; Eshkol *et al*., 2009; Fialho *et al*., 2011). Unlike plant weeds, where phytotoxic inhibitors tend to have the greatest impact on neighbouring plants, microbes can also produce volatile organic compounds (VOCs) that inhibit the growth of both proximal and distal cells via their dispersion in both aqueous and gaseous milieu (Table 6).

Volatile organic compounds – including aldehydes, furones, terpenes and many alcohols – can be powerful inhibitors of microbial metabolism and growth (Table 6; Jorge and Livingston, 1999; Passoth *et al*., 2006; Minerdi *et al*., 2009; Fialho *et al*., 2011; Corcuff *et al*., 2011). Despite their volatility they are to some degree also water-soluble, and are sufficiently inhibitory to prevent metabolic activity and growth, even in aqueous solution at subsaturated solute-concentrations (Bhaganna *et al*., 2010). VOCs with a log P_octanol-water_ < 1.95 typically partition into the aqueous phase of cellular macromolecules and can act as potent chaotropic stressors (Table 6; Hallsworth *et al*., 2003a; Bhaganna *et al*., 2010; Cray *et al*., 2013). Those stressors with a log P of > 1.95 preferentially partition into the hydrophobic domains of membranes and other macromolecular systems, and thereby act as hydrophobic stressors that induce a distinct type of chaotropicity-mediated water stress (Bhaganna *et al*., 2010). We believe that the primary ecophysiological value of VOCs for microbial cells pertains to competition, species succession and (for weed species) habitat dominance. *Saccharomyces cerevisiae* synthesizes both hydrophobic and chaotropic volatiles that have antimicrobial activities, including ethyl octanoate and isoamyl acetate, and ethyl acetate and 2-phenylethanol (respectively, see Table 6; Fialho *et al*., 2011). Ethanol and 2-phenylethanol are synergistic in their antimicrobial activity (Ingram and Buttke, 1984; Seward *et al*., 1996), and this is consistent with their common chaotropicity-mediated mode-of-action. The more potent chaotrope of the two (when compared on a molar basis) is 2-phenylethanol which prevents growth of microbes in the range 10–40 mM (Berrah and Konetzka, 1962; Eshkol *et al*., 2009), values consistent with a chaotropic mode-of-action for a stressor with a log P of 1.36 (see Bhaganna *et al*., 2010). *Saccharomyces cerevisiae* (like *P. anomala, Acinetobacter* and lactic acid bacteria) also inhibits the growth of competing species by acidifying the environment which it achieves via the production of a number of organic acids (Table 6; Kotyk *et al*., 1999; Lowe and Arendt, 2004; Gulati *et al*., 2010); it also secretes peptides with fungicidal and fungistatic activities (Albergaria *et al*., 2010); and some strains contain inheritable virus-like particles containing genes that code for proteins inhibitory to other strains of the same species (Pretorius, 2000). Not only does *S. cerevisiae* employ multiple types of antimicrobial substance that act in concert, it is also has a correspondingly wide range of stressor-protection measures that minimize metabolic inhibition by antimicrobial stressors (those synthesized by either *S. cerevisiae* or other species), including efflux pumps (Pretorius, 2000), and highly effective compatible-solute strategies (see above), protein-stabilization proteins and adaptations to acid stress (Table 6) and is therefore highly tolerant towards chaotropic and hydrophobic stressors (Fig. 2; Tables 4 and 6). *Saccharomyces cerevisiae* acid tolerance (as well as that of lactic acid bacteria) is conferred by an array of mechanisms (Table 3) that collectively contribute to the weed phenotype by removing the hydrogen ions and/or by promoting the refolding of partially denatured biomacromolecules.

*Pichia anomala* utilizes an array of antimicrobials that have synergistic effects as inhibitors and collectively target a wide array of taxa (including fungi, other yeasts, bacteria and viruses; Walker, 2011): chaotropic stressors such as ethanol and ethyl acetate; hydrophobic stressors such as isoamyl acetate; biosurfactants (see below); organic acids; an exo-β-1,3-glucanase known to degrade glucans in fungal cell walls; and diverse toxins that have specific modes-of-action (see Table 6; Passoth *et al*., 2006; Walker *et al*., 2011). A semi-quantitative analysis of the antifungal activities of *P. anomala* suggests that inhibition results primarily from a combination of (in order of importance): killer toxins, hydrolytic enzymes and volatile substances (Table 6), as well as more minor factors such as nutrient competition and substrate acidification (Walker, 2011). *Rhodotorula mucilaginosa* produces an array of volatile chaotropic and hydrophobic stressors as well as killer toxins (Table 6). Many fungi also produce mycotoxins; for example *Aspergillus ochraceus* growing on maize grain can produce ochratoxins at up to 1.4 mg g^−1^ (Magan *et al*., 2003). Indeed a number of *Aspergillus* species are notorious for the potent activity of their ochratoxins against other fungal strains and as well as bacteria (Table 6).

Like *S. cerevisiae*, some other microbial species are able to produce moderately chaotropic antimicrobials in bulk quantities: *Clostridium acetobutylicum* produces high concentrations of butanol, ethanol and acetone in anaerobic habitats, and *Zymomonas mobilis* produces high concentrations of ethanol (Tables S1 and6). Butanol is a particularly potent chaotrope that is highly inhibitory at millimolar concentrations (Bhaganna *et al*., 2010; Ezeji *et al*., 2010; Cray *et al*., 2013). We suspect that *Clostridium* spp. that can dominate chaotropic environments such as ammonia-rich animal slurries (Table S1) have an unusually high resistance to the net chaotropicity of the ammonia, ethanol, butanol, acetone and other chaotropic substances in their habitat (a hypothesis that has not yet been investigated to our knowledge; Williams and Hallsworth, 2009; Cray *et al*., 2013). The chaotropicity of urea and ammonia is greater than that of ethanol when compared at equivalent molar concentrations (Hallsworth *et al*., 2003a; Williams and Hallsworth, 2009) and there is evidence that some bacteria that secrete these compounds, including *Clostridium aminophilum* and *Pseudomonas stutzeri*, benefit from chaotropicity-induced inhibition of their competitors (Table 6). There is no absolute resistance to the inhibitory/lethal activities of chaotropic and hydrophobic stressors (Sikkema *et al*., 1995; Hallsworth, 1998; Hallsworth *et al*., 2003a; 2003b; 2007; Duda *et al*., 2004; Bhaganna *et al*., 2010; Bell *et al*., 2013) so the production of such antimicrobials coupled with reasonably effective self-protection mechanisms (see Tables S1 and6) can sometimes be the ultimate means to achieve community dominance (see also Table S1).

*Pseudomonas putida* can generate and/or secrete diverse antimicrobial substances including catabolites produced from degradation of macromolecular substrates, VOCs and biosurfactants (Table 6). Biosurfactants that solubilize hydrocarbons and other hydrophobic substrates can also be highly inhibitory to other microbial species. Studies of *Pseudomonas* rhamnolipids suggest a key role in competitive ability (Wecke *et al*., 2011); surfactants not only disorder plasma membranes and solubilize lipids, but can be chaotropic even for macromolecules that lack hydrophobic domains (Table 2; Cray *et al*., 2013). The membrane-permeabilizing activities of rhamnolipids are particularly detrimental to Gram-positive bacteria, and are lethal to *Phytophthora* zoospores (Kruijt *et al*., 2009, Wecke *et al*., 2011). Bacteriocins produced by pseudomonads, like those of lactic acid bacteria, modify cellular membranes increasing permeability, causing leakage, disrupting transport processes and causing a dissipation of proton-motive force (Table 6). Catabolites such as phenol, catechol, vanillin, biphenyl, and sodium benzoate that are produced from the degradation of high-*M*_r_ hydrocarbons have inhibitory activity as chaotropic and hydrophobic stressors (Table 6; Hallsworth *et al*., 2003a; Bhaganna *et al*., 2010). *Pseudomonas putida* and other pseudomonads produce a wide variety of VOCs that collectively impact microbial competitors (Table 6). As outlined above, *P. putida* has multiple layers of defence against the potential stressful impact of solvent, chaotropic, and hydrophobic stressors via a versatile compatible solute metabolism (e.g. trehalose; Park *et al*., 2007; Bhaganna *et al*., 2010), protein-stabilization proteins, and production of extracellular polymeric substances etc. (Tables 3 and 6). In addition, and arguably the most important feature for stressor tolerance, *P. putida* cells are equipped with efflux pumps (for example the plasmid-encoded TtGHI) that are highly effective at expulsion of hydrocarbons such as toluene as well as many other stressors and toxins (Ramos *et al*., 2002; Fillet *et al*., 2012).

Whereas the aquatic habitats of planktonic species such as *D. salina, G. semen* and *M. aeruginosa* may serve to dilute secreted antimicrobials, there is evidence that VOCs produced by *D. salina, M. aeruginosa,* and other aquatic species (including compounds such as β-cyclocitral and dichloromethane) are inhibitory to competing species (Table 6). *Gonyostomum semen* is able to lyse the cells of competing algal species on contact although the mechanism by which it does so has yet to be resolved (Table 6); and *M. aeruginosa* synthesizes a diverse range of volatile antimicrobials including phenazines and phenazine derivatives (Table 6; Lifshits and Carmeli, 2012). Lactic acid bacteria, in addition to bacteriocins, produce significant quantities of organic acids and secrete a vast array of inhibitory VOCs (Table 6). The analysis of volatiles produced, primarily by *L. rhamnosus*, during the production of a ewe's-milk cheese (Table S1) gives an insight into the complexity of the microbial warefare that takes place in open habitats: 57 compounds were detected including terpenes, alcohols, ketones and esters (Randazzo *et al*., 2010) Although it is plausible that some of these substances exert specific toxic effects, all of them have the properties of either chao- or hydrophobic stressors in microbial cells (Table 6; Bhaganna *et al*., 2010), and their roles in habitat dominance has rarely received attention.

## Microbial weed ecology

### Open habitats

The concept of an open habitat is a fundamental, well-established principle in the fields of plant and animal ecology. For microorganisms, open habitats can be defined according to the diversity, interactions and community succession of their inhabitants in contrast to other types of microbial habitat that are defined by physicochemical characteristics, substrate type or nutrient concentration (Table 7). Hydrocarbons are the primary carbon substrate in approximately one-third of the open habitats listed in Table S1, and are generally derived from dense, non-aqueous phase liquids (e.g. oil) or plant metabolites secreted into the rhizosphere, phyllosphere or atmosphere (bioaerosols). A distinguishing feature of hydrocarbon-based systems is that they can remain perpetually open because, as dissolved hydrocarbons are assimilated, the substrate reservoir may be continually replenished by molecules from the oil entering the aqueous phase or (for the rhizosphere and phyllosphere) because the plant continues to produce and secrete hydrocarbons and other organics (Nunes *et al*., 2005; Kazda and Falkinham III, 2009; McCammick *et al*., 2009; Hunter *et al*., 2010; Kachalkin and Yurkov, 2012). These habitats are commonly dominated by species such as *Acinetobacter johnsonii, Alkanivorax borkumensis, Pantoea ananatis* and *P. putida*. Whereas these organisms – all members of the gammaproteobacteria – may share many molecular and ecophysiological characteristics (see Tables 3) in most cases it is *P. putida* that emerges as the most prevalent species (Table S1). This is a product of its metabolic versatility, exceptional tolerance to hydrocarbons and other solutes, and antimicrobial activities (Tables 5 and 6). Other weed species, however, have greater acid tolerance than the gammaproteobacteria: in the acidic surface film of the sphagnum-moss grey layer it is *Mycobacterium* (including *Mycobacterium terrae*, *M. smegmatis* and *Mycobacterium sphagni*) that emerges as a dominant genus; and in the green layer at lower pH (2–2.5), *R. mucilaginosa* has the competitive advantage (Table S1). Phyllosphere, rhizosphere and other habitats can also remain continuously open if they are replenished with organic substances from ions released from soil particles, extracellular polymeric substances and compatible solutes from microbial cells, root diffusates, animal wastes, diverse sources of necromass, etc.

**Table 7:** Properties of open (microbial) habitats

		Notes and examples	
Typical characteristic(s)	Value as a descriptor for an open habitat^a^	For open habitats	For habitats that are not open
**Microbial ecology**			
Open to population by a multitude of microbial species	Primary	Potentially habitable by an indefinite number of species which may be from diverse Kingdoms of life (e.g. solar salterns see Table S1)	The fracture water of a South-African goldmine (2.8 km deep) was found to contain a single-species ecosystem (Chivian *et al*., 2008)
Promote intense competition between microbial species	Primary	Conditions favour strong growth of many species and may select for ability to produce antimicrobial substances (see below)	A climax community, single-species habitat, and symbiotic partnership are characterized by low levels of inter-species competition
Can facilitate dominance of the developing community by one or more species (though this may not always take place)	Primary	Diverse types of substrate/environment can accommodate community progression towards dominance by one or more microbial species (see Table S1)	The antithesis of an open habitat is one that is occupied by a climax community that is no longer capable of further evolution (e.g. Paul *et al*., 2006)
Transient and self-terminating; unless maintained in an ‘open’ condition	Primary	Frequently leading to the development of a closed community dominated by a single species (see Fig. 1; Table S1); unless ‘re-opened’ due to grazing for example, or replenished (e.g. by fresh inputs of nutrients)	Closed habitats typically lack conditions that facilitate biomass formation and species succession
**Resident microbes**			
Strong selection for microbes able to out-grow competitors	Primary	For example, habitats rich in nutrients may favour species of bacterial copiotroph which may merge as dominating species if they possess other weed traits	A habitat with limited available resources would not would not support growth (the latter is associated with competition/species succession)
Open to manipulation by species able to modify environmental conditions and/or secrete antimicrobials	Primary	Dynamic biomass formation makes open habitats available for/vulnerable to manipulation by species that can introduce growth-limiting substances (Table6; Cordero *et al*., 2012)	In closed habitats the community is already restricted in terms of space, nutrients and/or physicochemical constraints etc.
Select for microbes that lack specific requirements (such as rare trace elements, or a specific host)	Secondary	Microbes such as *S. cerevisiae* and *Lactobacillus* spp. typically grow without idiosyncratic requirements (such as a requirement for interactions with other species)	Specialist microbes are found in niche habitats, such as those that form lichens (Hoffland *et al*., 2004)
**Chemistry and properties of the cellular environment**			
Not excessively stressful for resistant microbes in terms of water activity, chaotropicity, pH, temperature etc.^b^	Primary	Open (microbial) habitats are, by definition, open to population by a large number and high diversity of microbial species so are typically not physically or chemically extreme^c^	Extreme habitats that are occupied by a small number of obligate extremophiles rarely support the high growth rates or promote the intense competition that typify open habitats^c^
Have the potential to support a substantial microbial biomass and so do not constrain ecosystem development	Primary	Open habitats have sufficient space and resources to facilitate the ecosystem development (Table S1; Fig. 1)	Non-open habitats can be constrained by nutritional and/or physicochemical parameters (Chivian *et al*., 2008)
Not contaminated with inhibitory substances such as heavy metals or other potent toxins	Secondary	Some polluted, hydrocarbon-rich environments may also support the development of microbial communities	High levels of toxic substances will prevent high growth rates and minimize diversity

a Those designated as descriptors of primary importance are considered essential to the definition of an open (microbial) habitat.

b Not so extreme that there is limited diversity or possibility for biomass formation: i.e. typically > 0.75 water activity (see Pitt, 1975; Williams and Hallsworth, 2009), < 20–25 kJ kg^−1^ mole^−1^ chaotropic activity (Hallsworth *et al*., 2007), < pH 11 or > −15°C.

c Whereas NaCl-saturated habitats are generally considered extreme, their water activity is ^3^ 0.75 – well above that which ultimately limits microbial life (i.e. 0.61–0.71: Pitt and Christian, 1968; Brown, 1990; Grant, 2004; Williams and Hallsworth, 2009; Leong *et al*., 2011) – and typically supports a remarkably high microbial diversity and biomass (Antón *et al*., 2002; Daffonchio *et al*., 2006; Baati *et al*., 2008; Bardavid *et al*., 2008; Khemakhem *et al*., 2010).

Habitats which facilitate weed emergence are typically not so extreme that there is a negligible microbial diversity or little capacity for community development/biomass formation; i.e. > 0.75 water activity, < 20–25 kJ kg^−1^ mole^−1^ chaotropic activity, < pH 11 or > −15°C (Table 7). Open habitats have available nutrients, electron acceptors etc and C : N ratios that favour microbial growth, and do not contain high levels of inhibitory substances such as heavy metals or toxic pollutants. They will therefore promote intense competition between species assuming that a diversity of microbial species is present (Tables S1 and7). Whereas high-sugar habitats with a water activity below 0.9 are too stressful for the vast majority of microbes, including *S. cerevisiae*, there is still a considerable diversity of xerotolerant and xerophilic species that are highly active between water activities of 0.84 and 0.9 (Pitt, 1975; Brown, 1990). Similarly, crystallizer ponds or solar salterns are known as extreme environments and only a small fraction of microbial species can survive and grow at saturated NaCl. Nevertheless, it is the limited solubility of NaCl (and not the limitations of salt-adapted macromolecular and cellular systems) that determines the limit for microbial growth in high-NaCl habitats: a number of obligate halophiles grow optimally at 35% w/v NaCl (0.755 water activity) and different types of NaCl-saturated environment are biodiverse, highly competitive, biomass-rich microbial habitats (Antón *et al*., 2002; Daffonchio *et al*., 2006; Baati *et al*., 2008; Bardavid *et al*., 2008; Khemakhem *et al*., 2010). Salterns (and also algal mats) may under some conditions function as continually open habitats if nutrients are not limiting; *D. salina* for example can release sufficient glycerol into the system to satisfy the carbohydrate requirement of the wider community (Borowitzka, 1981; Bardavid *et al*., 2008).

Open habitats favour microbes with shorter generation-times and relatively vigorous growth-phenotypes and in this way may drive the evolution of faster-growing and more stress-tolerant species (as well as those with effective antimicrobial strategies; Tables 6 and 7). That the ecology and evolution of plant weeds are intimately and inextricably linked with open habitats has been established for some time (Anderson, 1952); in contrast there is little information about open habitats of microorganisms (see Table 1; Hallsworth, 1998). Open habitats can be spontaneously created (e.g. due to removal of microbial biomass by predators and grazers; McArthur, 2006), dynamic (e.g. due to desiccation–rehydration cycles or nutrient limitation), and/or transient (Tables S1 and7). As is true for open habitats of plants (Anderson, 1952), open habitats of microbes can also become closed if they becomes water-constrained or otherwise resource-depleted; and/or the microbial community reaches stationary phase (Paul *et al*., 2006): and these changes may be accompanied by – or even a consequence of – domination by weed species. Environments can even progress to become sterile; for example those with high ethanol concentrations (Hallsworth, 1998; Pretorius, 2000). However, a dualistic concept of open habitats on the one hand and closed habitats on the other would be simplistic; in reality there is a seemless continuum between the two extremes of fertile but uncolonized and hostile/uninhabitable environment. Habitats with a favourable water activity for most bacteria (≥ 0.94) and high concentrations of carbon and nitrogen substrates such as milk, cheese and crushed peanuts (Table S1) typically progress to a closed condition (and are dominated by lactic acid bacteria), as do most high-sugar habitats (Table S1). A number of the open habitats listed in Table S1 that are resource-rich, and stressful, temporally or spatially heterogenous, and/or require saprotrophic nutrition were dominated by *Pichia* (e.g. moist barley grains; salted fermenting olives) or *Aspergillus* species (e.g. decomposing leaf litter, sugarcane juice). The stress biology, energy-generating capabilities and antimicrobial activities of these species undoubtedly contribute to their success (Tables 3 and 6).

### Ecophysiological classification

Microorganisms have been previously categorized based on the way in which their phenotype impacts ecological behaviour: as autotrophs and heterotrophs; generalists and specialists; *r*- and *K*-strategists, aerobes and anaerobes; copiotrophs and oligotrophs; fast and slow growers; and extremophiles and mesophiles (see Table 4 and S2). The definition of microbial weeds is qualitatively distinct from that of other microbial groups (see Table S2): only weeds are characterized by their ability to dominate communities and the associated/underlying biology (Tables 2) and it is only weeds that are linked via their ecology and evolution with open habitats (Tables 1, S1, and S2). The biology and ecology of microbial weed species cuts across established groupings of microorganisms (i.e. the former does not represent a parallel category; see Table S2). Some microbes have a considerable number of weed traits but only rarely emerge as dominant members of communities (e.g. *Pseudoxylaria* spp., *E. coli* and *M. smegmatis*; see Tables S1 and5 and 6). Such species serve as a reminder that weeds and non-weeds represent two extremes of an otherwise continuous scale. An insightful study of *Termitomyces* ecology, for example, discusses the biology of *Pseudoxylaria*; a fungus that is present in *Termitomyces* habitats but is typically inactive and/or present at negligible cell densities (Visser *et al*., 2011). *Pseudoxylaria* could be ecologically disadvantaged if present in greater numbers as termites, which can detect VOCs produced during interactions between *Pseudoxylaria* and *Termitomyces*, remove the unwanted fungus from their *Termitomyces* cultures. The extant *Pseudoxylaria* strains thereby appear to have been selected for/have evolved a low competitive ability (Visser *et al*., 2011). Nevertheless *Pseudoxylaria* can occasionally emerge to dominate the substrate (Visser *et al*., 2011), a fertile open habitat consisting of decomposing plant material (Table S1). Non-weed species make up a loose affiliation of species that are only linked by their poor ability to dominate communities in open habitats and may not share any specific metabolic or phenotypic traits (Table S2). We propose that this ‘dustbin’ group includes *Synodropsis* spp., *Polypaecilum pisce*, *Metschnikowia orientalis, Caulobacter crescentus* and *Salmonella* species (Table S2). Several ecophysiological classification systems that are based on microbial growth-phenotype and/or substrate utilization (Table S2). Microbial generalists can occupy a wide range of ecological niches, and typically achieve this via the ability to utilize a wide range of nutrients and substrates (Table S2). Conversely microbial specialists, such as *S. cerevisiae*, *H. werneckii* and many basidiomycetes have specific nutritional and/or physicochemical requirements (de Fine Licht *et al*., 2005; Kashangura *et al*., 2006). Obligate extremophiles commonly occupy, and are adapted to grow optimally in, niches that are hostile to the majority of microorganisms (Table S2). There have been relatively few studies (and is consequently inadequate terminology) to properly describe microbes that are extremo-intolerant such as xero-intolerant species (see Table 4), with the exception of mesophilic species that function optimally at non-extreme temperatures (though the term *mesophiles* can be used in a general sense to refer to non-extremophiles: Table S2; Gostinčar *et al*., 2010). Non-extremophilic microbes grow optimally in physicochemical conditions which favour metabolic activity and growth of the majority of microbial species, and this grouping includes both weed and non-weed species (Table S2). While fast-growing microbes have short doubling times, the vast majority of these species do not have weed-like capabilities and are unable to out-grow their competitors (Tables 4 and S2). It may be that relatively few oligotrophic microbes or *K*-strategists are weed species, although some mycobacteria do have a weed phenotype (Tables S1,5 and 6). *Pseudomonas putida* is generally considered to be a model *r*-strategist (e.g. Margesin *et al*., 2003) yet *P. putida* has an extraordinary competitive ability (see Tables S1 and6), and may not be the best example of an *r*-strategist (the latter are characterized by a weak competitive ability; Table S2). By contrast *P. putida* appears to be an exceptional example of a microbial weed both in terms of its cellular biology and its ability to dominate diverse types of open habitat.

## Concluding remarks

It is irrefutable that a small minority of microbial species recurrently appear as the dominant members of microbial communities; and we suggest here that these microbes have shared phenotypic traits. This implies that open-habitat ecology has impacted the evolutionary trajectories of these microbes, and that the activities of weeds determine microbial diversity in many localities within the biosphere. Whereas microbial weed species are not always useful to humankind (see Table 1), they make up a substantial portion of the world's biotechnology sector. *Saccharomyces cerevisiae* supports global industries in fermented products, biofuel, biocides (ethanol is also an important disinfectant; McDonnell and Russell, 1999), and other white biotechnologies; *P. anomala* produces a substantial catalogue of biotechnologically useful substances and is employed in production of foodstuffs (see Walker, 2011); lactic acid bacteria form the basis of food- and drinks-production processes where the antimicrobial volatiles and organic acids that they secrete act as flavour compounds and natural preservatives, and are used as probiotics, and for the production of fine chemicals (Rose, 1982; Steinkraus, 1983; Molly *et al*., 1996; Nomura *et al*., 1998; Tiwari *et al*., 2009; Randazzo *et al*., 2010; Bron *et al*., 2012); *P. putida* is used for the commercial production of bulk and fine chemicals, industrial biocatalysis, and as the basis of many other industrial and environmental biotechnologies (Kruijt *et al*., 2009; Puchałka *et al*., 2008; Hassan *et al*., 2011; Poblete-Castro *et al*., 2012); a number of weed species, including *Aspergillus* spp., *P. putida* and *Clostridium* spp. play primary roles in the treatment or decomposition of plant matter, oil and hydrocarbons, xenobiotics in polluted soils, sludge and slurry treatments, and other organic wastes (Tables S2 and4; Narihiro and Sekiguchi, 2007; Timmis, 2009); and *Aspergillus* species are used for production of foods (Rose, 1982; Tamang, 2010), fine chemicals (e.g. citric acid, secondary metabolites) and extracellular enzymes (e.g. amylase, cellulase, pectinase and protease), for industrial biocatalysis, and have potential for bioremediation and biosorption applications (Ward *et al*., 2006). It is the metabolic activities of substances that suppress potential competitors which make *S. cerevisiae*, *Rhodotorula* spp., *P. anomala*, *P. putida*, lactic acid bacteria and other weed species invaluable as agents of biological control, for preventing spoilage of drinks, foodstuffs and animal feeds (see Calvente *et al*., 1999; Kruijt *et al*., 2009; Hassan *et al*., 2011; Olstorpe and Passoth, 2011; Walker, 2011).

Microbial weeds are also used as model systems for research, yet key aspects of their biology remain enigmatic. Many plant weeds have exceptional reproduction and dispersal systems (Table 1) and it remains unclear whether microbial weed species produce more propagules, have superior dispersal mechanisms, or evolved propagules with exceptional longevity, and/or the ability to germinate over a wider range of conditions than their non-weed comparators. Could it be that, like plant weeds, the competitive ability of microbial weeds is enhanced by an abnormally high phenotypic plasticity and intra-specific variability (see Table 1)? Out of the world's plant flora it has been estimated that only several hundred plant species are weeds (Hill, 1977); what percentage of microbes might exhibit weed phenotypes and behaviour? Is there evidence preserved in rocks, sediments, peat, ice, or the hypersaline fluid inclusions of salt crystals that can shed light on the evolutionary trajectories of microbial weed species? Is there an ideal genome size for prokaryotic and eukaryotic weed species? What are the minimum/essential characteristics required to enable a microorganism to dominate a specific habitat; can we design and manufacture a microbial weed via a synthetic biology approach? Will hitherto uninvestigated microbial weeds prove to be rich sources of antimicrobial compounds for novel biotechnological and clinical applications? Can microbial weed ecology shed light on wound microbiology, lead to the prevention of the microbial colonization of prosthetic devices, or minimize the emergence of opportunistic pathogens? Could knowledge-based interventions in weed ecology enhance our ability to manipulate and manage microbes at various levels of food-production and food-spoilage, or lead to better management of environments that harbor potentially dangerous species?

Crop plants such as sunflowers, potatoes, wheat and barley are known to have weedy ancestors (Anderson, 1952) and it is thought that the evolution of both plant weeds and crop plants has been driven by anthropogenically opened habitats. We believe that open (microbial) habitats also select for the phenotypes, and therefore act as a potent driving force that determines the evolutionary trajectories, of microbial weeds and their communities: open-habitat ecology may therefore be worthy of recognition as a distinct scientific field that spans all organismal biology including microbiology. There is a maxim that everything is everywhere but that the environment selects; this implies that microbial species are everywhere on Earth but that the local environment determines which are able to grow (Baas Becking, 1934; de Wit and Bouvier, 2006). While not necessarily untrue, this idea implies a level of passivity on the part of the microbial cell. Yet microorganisms are inherently variable and dynamic systems with considerable genetic dexterity and phenotypic plasticity (able to both respond and adapt). Baas Becking (1934) acknowledged that cells are able to modify their extracellular environment, but microbes are also proactive in their manipulation of each other. It may be that microbial weeds are the most accomplished examples of this.

## Supporting information

**Table S1**. Examples of open habitats in which specific microbes attain dominance. **Table S2**. Biology of microbial weeds compared with that of other ecophysiological groups.
