# Supplementary material for: The biology of habitat dominance; can microbes behave as weeds?
Source: Microb Biotechnol. 2013 Jan 22;6(5):453–92. doi: 10.1111/1751-7915.12027 (PMC3918151; doi:10.1111/1751-7915.12027)
Supplement: Supplementary file 1 — Table S1. Examples of open habitats in which specific microbes attain dominance. Table S2. Biology of microbial weeds compared with that of other ecophysiological groups. [file mbt0006-0453-sd1.doc]

**Supporting Information**

**Contents**

- Table S1
- Table S2
- Supporting References

**Table S1.** Examples of open habitats in which specific microbes attain dominance.^a^

_____________________________________________________________________________________________________________________________

Habitat^b^ Physical, chemical and/or nutritional Examples of genera present^c^ Additional notes and references conditions

_____________________________________________________________________________________________________________________________

**Atmosphere**

*Bioaerosols above* Bioaerosols above the rainforest are ***Acinetobacter***, *Bacillus,* The microbial communities of bioaerosols can have

*the Amazonian* characterized by high concentrations of *Pseudomonas, Xanthamonas*, high rates of metabolic activity and cell division

*rainforest*^d^ organic substances (Nunes *et al*., 2005) and other genera (Womack *et al*., (Womack *et al*., 2012). *Acinetobacter* has also been

2012) identified as a prevalent species in other types of

bioaerosol (e.g. Hameed and Khodr, 2001)

*Bioaerosols above* Carbon-, nitrogen- and phosphate- *Deinococcus, Propionibacterium*, Eight culture-independent analyses that were carried

*the Baltic Sea*^d^ concentrations were apparently sufficient ***Pseudomonas***, *Psychrobacter,* out during a 12-month sampling period identified

to sustain microbial growth ***Sphingomonas*** *Pseudomonas* and *Sphingomonas* spp. as the

prevalent microbes (up to 61 and 44% of the bacterial

community, respectively; Fahlgren *et al*., 2010)

**High-salt**

*Saltern crystallizer* NaCl = 34% w/v; water activity ~ 0.755; *Halonotius*, *Haloplanus*, *Haloquadratum walsbyi* commonly occurs as the

*ponds* average annual rainfall = 681 mm; aver- ***Haloquadratum***, *Halorubrum*, dominant species in NaCl-saturated environments

age annual maximum and minimum temp- *Natronomonas*, and other genera (Oh *et al*., 2010; Ghai e*t al*., 2011) that are typically

eratures = 28 and 17°C respectively biodiverse, high biomass, and highly competitive

microbial habitats (Daffonchio *et al*., 2006 ; Baati *et*

*al*., 2008; Khemakhem *et al*., 2010)

Salt concn. ~saturation; average annual *Chlamydomonas,* ***Dunaliella****,* Species identification was carried out for a diverse

maximum and minimum temperatures of ***Haloquadratum***, *Navicula,*  microbial community in a Tunisian solar saltern

~37 and 15°C respectively. Total nitrogen *Nitzschia, Oxyrrhis,* ***Salinibacter***, (*TS* saltern) over a 4-year period (Baati *et al*., 2008;

≤ 38 μmol l^-1^; total phosphate ≤ 56 μmol l^-1^ other Archaea (within the Khemakhem *et al*., 2010). Phytoplankton such as

(Baati *et al*., 2008; Khemakhem *et al*., Euryarchaeota), and bacteria *Navicula, Nitzschia* and *Oxyrrhis* are less salt-

2010) (within the Bacteroidetes and tolerant than *Dunaliella* (Khemakhem *et al*., 2010).

Euryarchaeota) *Dunaliella salina, Salinibacter ruber* and *H. walsbyi*

frequently occur together as the dominant species in

hypersaline environments (Antón *et al*., 2002;

Bardavid *et al*., 2008)

*Olive fermentation* NaCl (6% w/v) + lactic acid (0.2% v/v); *Aureobasidium*, *Candida*, Sampling times were 2, 17 and 35 days; *Pichia*

20°C; initial pH 6.2; final pH 3.6 *Cystofilobasidium*, *Debaryomyces*, species were undetected after 2 days but comprised

*Metschnikowia*, ***Pichia***, ≥ 87% of the community by 17 and 35 days (Nisiotou

*Rhodotorula*, *Saccharomyces*, *et al*., 2010; see also Fig. 1C)

and diverse lactic acid bacteria

(genera not identified)^e^

*Ewe’s-milk cheese* NaCl = 7.1–9.7% w/w; water activity = *Enterococcus*, *Lactococcus*, In the curd there was a high diversity of lactic acid

*(Pecorino Croton-* 0.95–0.85; pH 5–5.25 ***Lactobacillus***, *Leuconostoc* bacteria; the most prevalent species were *Enteroco-*

*ese)*  *Streptomyces* *ccus faecalis* (at 35%) and *Leuconostoc mesenteroid-*

*es* (26%). Cheese samples were taken at 60 and 120

days by which time communities were dominated by

*Lactobacillus rhamnosus* (56–81% of the community;

Randazzo *et al*., 2010)

**Marine**

*Coral reefs* Fungi were isolated from diverse surfaces *Acremonium, Alternaria,* ***Aspergi-*** Evidence suggests that these fungi are active in the

in Australian coral-reef systems (e.g. ***llus***, *Cladosporium, Cochliobolus,* coral-reef system (Morrison-Gardiner, 2002; Jones,

algae, invertebrates, and sediments; *Curvularia, Fusarium, Gilmaniella,* 2011)

Morrison-Gardiner, 2002) *Gliomastix, Humicola, Monilia,*

***Penicillium****, Phoma, Rhizopus,*

*Trichoderma, Zygosporium*

*Mixed-species* Bacteria were isolated from the surface of  *Alteromonas, Cytophaga,* The most prevalent bacterial species,

*biofilms on seaweed* a macroalga, *Ulva lactuca*, growing in the ***Pseudoalteromonas****, Roseo- Pseudoalteromonas tunicata*, was characterized by

intertidal zone (Sydney, Australia)  *bacter*, and other genera effective biofilm formation and the secretion of

antibacterial substances (Rao *et al*., 2005)

*Coastal upwelling* Mean ocean temperatures were 14°C ***Bathycoccus*, *Micromonas*** The three most-prevelant eukaryotic plankton (Collado-

*(Chile)* and the mean phosphate concentration ***Ostreococcus***, and members of Fabbri *et al*., 2011) have been the focus of numer-

~1–1.5 μmol l^-1^ the Crustomastix, Prasinococc- ous studies of their ecology and resistance to viruses

ales, Pyramimonadales, Trebo- (e.g. Thomas *et al*., 2011; Table 6)

uxiophyceae

*Arctic sea-ice* Samples taken from bottom ice at ~ 1.5 *Fragilariopsis, Navicula,* ***Nitzschia*** After four sampling events over a three-week period,

m; snow depth = 3–6.8 cm; particulate and other diatom groups *Nitzschia frigida* was found to make up between 76

organic matter = 231–865 mg m^-2^ and 91% of the microbial community (Michel *et al*.,

2002). Other studies also report the dominance of this

species (e.g. Haecky *et al*., 1998)

**Freshwater**

*Eutrophic and* Hypertrophic lake water *Anabaenopsis, Aphanizomenon,* This community analysis was carried out in samples

*hypertrophic rivers Limnothrix, Merismopedia,* from an urban lake in Porto (Portugal; Leão *et al*.,

*and lakes* ***Microcystis***, and members of the 2012)

Chlorophytes, Dynophytes,

Oscillatoriales

*Humic lakes* Typical temperature ~ 10°C; pH ~ 5.7; ***Gonyostomum*** and members of *Gonyostomum semen* is frequently the most prevalent

total nitrogen, phosphorous and organic the Bacillariophyceae, Chlorophy- species in plankton blooms in boreal brown-water

carbon = 522, 13.7, and 10.8 μg l^-1^ ceae, Chrysophyceae, Cryptophy- lakes (Trigal *et al*., 2011). A typical *G. semen* density

respectively ceae, Dinophyceae is 1.5–4.5 mm^3^ l^-1^ (Trigal *et al*., 2011)

**Rock and sediments**

*Oil-contaminated* Rocks were polluted with crude oil *Aeromonas*, *Alcanivorax,* At week 12 of this study the community was dominated

*rocks*  *Paracoccus,* ***Pseudomonas***, by *Pseudomonas* *putida* (prior to biostimulation via

and other genera addition of a liquid fertilizer; Ogino *et al*., 2001)

*Intertidal mudflat* Oil (70 g kg^-1^) and Osmocote® (a slow- ***Alcanivorax***, ***Pseudomonas***  Microbial diversity was assessed (by DGGE) and

*(supplemented with* release fertilizer containing equal parts of and other genera the dominating species by Day 11 identified as

*oil and slow-release* nitrogen, phosphate and potassium) were *Alcanivorax borkumensis* and *Pseudomonas stutzeri*

*fertilizer)* added (Röling *et al*., 2004)

*Sand microcosm* Phenanthrene = 200 mg kg^-1^, lyophilized *Alcaligenes*, *Enterobacter*, Microcosms were inoculated with bacteria from a

*(supplemented with* ryegrass-root exudate = 10 g kg^-1^; water- *Herbasprillum*, *Paenibacillus*, hydrocarbon-contaminated soil; community compos-

*phenanthrene and* holding capacity = 80%; temperature = ***Pseudomonas***, *Stenotropho-* ition was assessed after a 5-day incubation period

*root exudates)* 24°C  *monas* (Louvel *et al*., 2011)

**Soils**

*Rhizosphere of* Physicochemically heterogeneous *Agrobacterium*, *Arthrobacter*, The rhizosphere of 5-year-old seedlings of Gala

*apple trees* substrate with inputs from root exudates *Burkholderia*, *Cytophaga*, apples was dominated by pseudomonads (32% of

and products of saprotrophic microbial *Enterococcus*, *Flavobacterium*, species present); including *Pseudomonas fluorescens*

activity *Phyllobacterium*, ***Pseudomonas***, and *P. putida* that collectively made up 31% of the *Rhodococcus*, *Stenotrophomonas*, community (Mazzola, 1999) *Variovorax*

*Forest soil* Soil samples were obtained from a ***Armillaria***, and diverse genera of This habitat has constant inputs of organic matter

hardwood forest in northern Michigan, soil microbes (including numerous from root exudates; leaf litter, microbial and other

USA; typical temperature = 14°C (Smith saprotrophic fungi) sources of necromass, animal droppings etc; fungi are

*et al*., 1992) continually grazed by the soil fauna so it is effectively

a perpetually open habitat for saprotrophic macrofungi

*Soils adjacent to* Samples were taken from the soil surface ***Aspergillus****, Cunninghamella,* Eleven species of fungi (from three genera) were

*buildings, roads etc* and at depths of up to 30 cm *Microsporum* identified in soil communities (2336 colonies were

obtained from five sampling sites); the upper soil

profile was dominated by *Aspergillus niger* and

*Aspergillus flavus* (Irum *et al*., 2007)

*Hydrocarbon-* Soil adjacent to an industrial (polyethylene- *Agrobacterium*, *Alcaligenes*, Community composition was determined at two- to

*contaminated* production) plant contained: benzene, *Arthrobacter*, *Azospirillum*, *Bacillus*, three-week intervals; *Pseudomonas* spp. dominated

*soil*  cyclopentadiene, dicyclopentadiene, *Bacteroides*, *Bordetella*, *Clostrid-*  during a six-week period (Greene *et al*., 2000)

toluene, styrene, xylenes, naphthalene *ium*, *Desulfovibrio*, *Flavobacterium*,

etc *Microbacterium*, *Nocardioides*,

***Pseudomonas***, *Rhodococcus*,

*Sphingomonas*, *Xanthomonas*

**Polysaccharides**

*Decomposed humus* Decomposed leaf litter from the grass *Alternaria*, *Ascochyta*, ***Aspergillus***, The moist, high-nutrient conditions in the litter – as

*in tropical grassland* species *Cymbopogon caesius* growing in  *Aureobasidium, Phoma,* well as constant inputs of organic matter – create and

a moist, sandy loam *Pithomyces,* ***Rhizopus****,* maintain a relatively open habitat for saprotrophic

*Scytalidium, Spegazzainia,* microbes (Senthilkumar *et al*., 1993)

*Taeniolella,* ***Trichoderma***

*Barley grain* Temperature = ambient; pH = 6.0; water *Acremonium*, *Cryptococcus*, The microbial community on moist barley grain

activity = 0.83 *Enterococcus*, *Lactobacillus*, that had been stored for five months was dominated

*Leuconostoc*, ***Pichia****, Pediococcus*, by *Pichia* spp. (*Pichia burtonii* and *Pichia anomala*;

*Sporobolomyces*, *Weissella* Olstorpe *et al*., 2010)

*Maize dough* Initial pH = 5.8 (final pH = 4.5); initial *Bifidobacterium, Enterococcus*, Sampling was carried out throughout a 96-hour

ethanol concn. = zero; final ethanol *Exiguobacterium*, *Lactobacillus*, period. Species such as *Exiguobacterium acetylicum*

concn. = 0.78% w/w *Leuconostoc*, *Oxalophagus*, and *Oxalophagus* sp. were initially prevalent but the

***Streptococcus****, Weissella,* and community was ultimately dominated by

diverse yeast and fungi; ~ 20 *Streptococcus* (ben Omar and Ampe, 2000)

genera detected (but not identified)

*Cauim (rice and* Crushed rice and crushed peanuts (cooked *Bacillus*, *Candida*, Inoculated using mastication juice of sweet potato.

*peanut) fermentation* in water); prevalence of lactic acid bacteria *Corynebacterium*, members of The community was initially biodiverse but by 48 h

suggests a low pH Enterobacteriaceae^f^, *Bacillus*, *Candida*, *Corynebacterium*, *Leuconostoc*,

*Kluyveromyces*, ***Lactobacillus***, *Rhodosporidium* and *Pichia* were no longer detectable.

*Leuconostoc*, *Pichia*, By contrast, *Lactobacillus* spp. dominated the

*Rhodosporidium*, *Saccharomyces* community (Ramos *et al*., 2010)

*Composting organic* At 12 hours the temperature was 24°C and ***Bacillus***, *Klebsiella*, *Lactobacillus,* There was no zero-time sampling point in this study.

*and food-waste* pH 5.6; by 96 hours these values were *Lactococcus*, *Pediococcus,*  By 24 hours (at 31°C) *Weissella* *confusa* was pred-

*(early-stage)* 44°C and 7.4 respectively *Pseudomonas*, *Weissella* ominant, but by 96 hours *Bacillus* spp. dominated the

community (Schloss *et al*., 2003^g^)

**Phyllosphere**

*Sphagnum moss* Typical pH of the surface film is 2–2.5; and *Aureobasidium, Candida, Cryptococcus* and *Rhodoturola* were the predominant

*(upper, green layer)* it contains a range of naturally occurring ***Cryptococcus****, Cystofilobasidium* yeast species in this habitat: up to 22 and 60% of the

sugars, amino acids, hydrocarbons, *Dothidella, Pichia,* ***Rhodotorula****,* total community, respectively. Percentage prevalence

phenolic compounds and other organics *Sporobolomyces* was based on colony counts (Kachalkin and Yurkov,

(Kachalkin and Yurkov, 2012; see also 2012)

Kazda *et al*., 2009)

*Sphagnum moss* Typical pH of the surface film is 4.5–5; and ***Mycobacterium***, diverse genera of Despite the high microbial diversity and biomass that

*(grey layer)* it contains products of the saprotrophic bacteria, fungi, microalgae, and typify this fertile (albeit acidic) habitat, diverse lines of

decomposition of moss as well as the other other groups evidence suggest that mycobacteria are typically a

substances found in the surface film of the prevalent/dominant genus (Nguyen-Viet *et al*., 2007;

green layer (see above) Kazda *et al*., 2009)

*Crofton weed* Analyses were carried out of healthy ***Acinetobacter****, Enterobacteriacea,* The dominant microbial group was the gamma-

(Ageratina leaves from the upper shoots of plants *Erwinia, Legionella, Methylibium,* proteobacteria; metagenomic analyses suggested

adenophora) growing at high altitude in Kunming, ***Pseudomonas****, Shigella,* that *Acinetobacter johnsonii* and *Pseudomonas* spp.

China  *Voriovorax*, and others including were the dominant microbes in this habitat (Zhou *et*  yeasts and fungi *al.,* 2010)

*Lettuce* Water content ≥ 90% w/w; soluble carbo- *Acinetobacter, Aeromonas,* Samples of lettuce leaf were macerated to extract

hydrate = 62.5 mg g^-1^ dry weight; phenol- *Buttiauxella, Enterobacter,* bacterial DNA for community analyses (Hunter *et al*.,

ics = 136.9 mg g^-1^ dry weight *Erwinia, Klebsiella, Pantoea,* 2010)

***Pseudomonas****, Rahnella, Serratia*,

and other genera

*Rice seedlings* Physicochemical conditions and available *Caulobacter, Methylobacterium,* Culture-independent techniques were used to analyze

*(grown* in vitro*)* nutrients were not determined  *Micrococcus*, ***Pantoea*** the phyllosphere community of 8-day old seedlings

(Kaga *et al*., 2009). *Pantoea ananatis* can dominate

other microbial habitats (De Baere *et al*., 2004; Coutinho

and Venter, 2009)

*Mature rice plants* Bacteria may access some soluble  *Aurantimonas, Bacillus,* Endophytic communities of 120 to 140-day plants of

*(Uruguay)* nutrients as well as vapour-phase hydro- *Curtobacterium, Microbacterium,* three rice cultivars grown in the field over two growing

carbons within the leaf  *Methylobacterium,* ***Pantoea***, seasons were analyzed; 51% of isolates were identif-

***Pseudomonas****, Sphingomonas,* ied as *P. ananatis* or *Pseudomonas syringae*

*Staphylococcus, Xanthomonas* (Ferrando *et al*., 2012)

**High-sugar**

*Sugarcane juice* The primary solute is sucrose; water *Absidia, Acremonium,* ***Aspergillus****,* The low water activity of sugarcane juice is close to or

activity in the range ~ 0.85 to 0.90 *Curvularia, Fusarium, Gilmanieila,* beyond the growth window of *S*. *cerevisiae* (see

*Humicola, Monilia, Penicillium,* Hallsworth, 1998) but selects for vigorous and

*Rhizopus, Saccharomyces* competitive species of xerotolerant fungi, especially

*A. niger* (Ahmed *et al*., 2010)

*Grape must* pH ~ 4.5 at Day 0; glucose + fructose *Debaryomyces*, *Dekkera*, The initial low pH favours acid-tolerant species. Rapid

concn. was ~ 10.8% at Day 0, 0.8% by *Kloeckera, Lactobacillus*, sugar utilization and ethanol synthesis by

Day 21 and 0.5% w/w by Day 84; ethanol *Leucosporidium, Rhodotorula*, *Saccharomyces* enables domination (de Pina and

concn. was 1% at Day 0, 3% by Day 21 ***Saccharomyces****, Torulaspora* Hogg, 1999). During fermentations carried out by

and 4% w/w by Day 84 *Zygosaccharomyces*^h^ indigenous microflora, ethanol-tolerant *S. cerevisiae*

strains invariably come to dominate (Pretorius, 2000)

*Apple must* Initial ethanol concn. = zero; final ethanol *Brettanomyces, Hanseniaspora,* Sampling was carried out over 36-day period. Most sugar

concn. = 10–11% v/v *Metschinikowia, Pichia,* utilization and ethanol production took place between

***Saccharomyces****,* 2 and 10–16 days (Morrissey *et al*., 2004)

*Saccharomycodes*

*High-glucose* Interaction studies carried out on malt- *Basipetospora*, ***Aspergillus***, The dominating species were generally *Aspergillus*

*nutrient media*  extract, yeast-extract phosphate agar ***Eurotium***, *Polypaecilum wentii* and *Eurotium rubrum*, species known to

(MYPiA) over a range of glucose concns. dominate communities on high-NaCl media and/or

from 0.98 to 0.84 water activity (at 15 to dried salted fish (Wheeler and Hocking, 1993)

30°C)

**Nitrogenous**

*Goat’s-milk cheese* Initial NaCl concn. was 1.73% w/v; NaCl *Enterococcus*, *Lactobacillus*, Samples were taken throughout the cheese product-

was added on Day 7 to give 6.84% w/v. ***Lactococcus***, *Leuconostoc*, ion process (4–8 months). *Lactococcus* achieved

Temperature = 20°C; initial pH unknown *Macrococcus*, *Mycobacterium*, domination within one week via a >5000 fold increase

but a gradual decrease occurred to pH 4.62 *Streptococcus* in cell number. This was 2.5Х greater than that of

*Lactobacillus* and 6.3Х greater than that of *Leucono-*

*stoc;* the second and third most prevalent genera

respectively (Casalta *et al*., 2009)

*Italian salami* Lard (45% w/w), NaCl (2.5% w/w), white *Brevibacillus*, *Debaryomyces*, Sampling was carried out at 1, 3, 10, 20, 30, and 45

*(Ciauscolo)* wine (9.5 ml kg^-1^) and crushed garlic (2.8% *Escherichia*, ***Lactobacillus***, days; *Lactobacillus plantarum* dominated by Day 3

w/w); temperature = 12°C; initial pH = 5.6; *Rhodotorula*, *Saccharomyces*, (by contrast *Saccharomyces cerevisiae* was no longer

final pH = 5.04; initial water activity = 0.94; *Staphylococcus*, *Trichosporon* detectable by this time; Aquilanti *et al*., 2007)

final water activity = 0.88^i^

*Pig slurry* Nitrogen content = 0.31–0.36% w/v; ***Clostridium***, *Escherichia*, The pig slurry may have been in the transition from

temperature = ambient; initial pH ~ 7 *Lactobacillus*, *Methanobrevibacter*, open to climax habitat prior to excretion from the

*Methanogenium*, *Porphyromonas*, animal and in the ~ 2-week period before sampling

*Streptococcus* commenced. The most prevalent organism was

putatively identified as *Clostridium botulinum,* and as

the sampling time increased (≥ 112 days) the proport-

ion of *C. botulinum* continued to increase (Peu *et al*.,

2006)

___________________________________________________________________________________________________________________

1. Generic properties of open habitats are described in Table 7.
2. The categories listed are not mutually exclusive.
3. Those that came to dominate the community are underlined in bold.
4. Bioaerosols are habitats in which microbes and their communities are metabolically active and undergo cell division (Womack *et al*., 2010).
5. See Nisiotou *et al*. (2010).
6. The marker used for identifying and quantifying Enterobacteriaceae pertained to family rather than genus.
7. This study did not include eukaryotic species.
8. Other yeast genera are commonly associated with grape must such as *Brettanomyces*, *Candida*, *Cryptococcus*, *Hanseniaspora*, *Kluyveromyces*,

*Metschinikowia*, and *Pichia* (Pretorius, 2000).

**i.** These water activity values suggest that the concentration of NaCl in the aqueous phase was considerably higher than 2.5% w/w.

**Table S2.** Biology of microbial weeds compared with that of other ecophysiological groups.^a^

____________________________________________________________________________________________________________________________________________________________________________

Microbial group Primary characteristic(s) Other traits Examples

____________________________________________________________________________________________________________________________________________________________________________

*Weeds*^b^ Thrive in, and dominate, microbial May out-grow competitors via efficient use of resources, a short *Acinetobacter* spp*.*, *Aspergillus*

communities that develop in open generation-time, tolerance habitat-relevant stresses, production *niger*, *Clostridium acetobutylic-*

habitats (see Fig. 1; Table S1) of antimicrobials, modification of the extracellular environment to *um*, *Dunaliella salina*, *Gonyost-*

create hostile conditions, and/or resistance to antimicrobial *omum* *semen*, *Haloquadratum*

toxins and stressors of biotic origin, etc (see Tables 4–6). Weed *walsbyi*, *Lactobacillus lactis*,

species are unlikely to have highly specific requirement(s) for *Lactobacillus rhamnosus*, *Mic-*

symbiotic, parasitic and/or microbial consortium interactions; *rocystis aeruginosa*, *Pantoea*

may be generalist, specialist, extremophilic, mesophilic etc (see *ananatis, Pichia* *anomala*,

Table S2); unlikely to have an extremely small or large genome *Pseudomonas aeruginosa*,

size *Pseudomonas putida*, *Rhodoto-*

*rula mucilaginosa*, *Saccharo-*

*myces cerevisiae*, *Salinibacter*

*ruber*

*Poor competitors* Inability/poor ability to dominate Few of the phenotypic characteristics that confer ability to *Caulobacter crescentus*,

*in open habitats* microbial communities that develop compete in open habitats (see Tables 3, and 5–7). May have a *Metschnikowia orientalis*,

*(non-weeds)* ^c^ in open habitats specialist lifestyle and/or be adapted to niches with an inherently *Polypaecilum pisce*, *Salmonella*

low microbial diversity spp., *Synedropsis* spp.

*Fast-growers* Short doubling-time; fast Unlikely to have specific requirement(s) for growth factors and *Debaryomyces* *hansenii*, growth in uncrowded conditions symbiotic, parasitic and/or microbial consortium interactions. *Escherichia coli*, *Lactobacillus*

Likely to have a small genome size^d^, and may compete via spp., *S. cerevisiae*

efficient use of resources

*Slow-growers* Long doubling-time; low maxim-May have poor competitive ability or alternative methods of Actinomycete spp.,

mum growth-rate competition (such as potent antimicrobials) *Mycobacterium szulgai*

*Copiotrophs*^e^ Thrive at high-nutrient concentr- Short doubling-time; rapid growth-rate. Compete via rapid *Escherichia coli*, many lactic

ations (especially in high-sugar use of resources and ability to withstand subsequent famine. acid bacteria, *P. putida*, *S.*

substrates) Growth-rate is typically determined by substrate richness^f^. *cerevisiae*, *Salmonella* spp.

Likely to have multiple copies of genes for and/or efficient

nutrient transporter proteins, and a small genome size

*Oligotrophs* Able to grow at minimal nutrient May be inhibited and readily out-competed at high-nutrient *Caulobacter crescentus*,

concentrations (see Koch, 2001) concentrations *Cycloclasticus oligotrophus*,

*Thermus aquaticus*

r*-strategists* Ability to proliferate (under specific Short lag-phase and high growth-rate; tendency to switch Many planktonic species includ-

conditions) in dynamic or unstable from growth to reproduction (e.g. sporulation) and/or die in ing some cyanobacteria and

environments, but otherwise have a crowded habitats (Andrews and Harris, 1986) dinoflagellates, *Salmonella* spp.

poor competitive ability

K*-strategists* Slow-growers that typically Resources are typically used for growth and maintenance *Agrobacterium* spp., many

populate stable/predictable – rather than reproduction – under crowded conditions basidiomycetes, *Coryne-*

habitats and have good compet- (Andrews and Harris, 1986) *bacterium* spp., *Mycobacterium*

itive ability spp., soil streptomycetes (Atlas

and Bartha, 1993; Wittmann *et*

*al*., 1998; Margesin *et al*., 2003)

*Generalists* Able to occupy a wide range of Ability to adapt to change. Likely to have a larger-than-average *Acinetobacter* spp*.*, *Aspergillus*

ecological niches by utilizing genome size due to nutritional versatility and/or genes involved spp., *Aureobasidium pullulans*,

a wide variety of nutrients/resou- in responses and adaptations to multiple stresses *P. anomala*, *P. putida*, *R.*

rces and/or tolerating a wide range *mucilaginosa*

of habitat-relevant stresses^g^

*Specialists* Can only occupy one (or a very Low metabolic versatility. Growth can be dependent on high *Hortaea werneckii*, *L. lactis*,

restricted number of) habitat types. concentrations of particular sugars (e.g., *S. cerevisiae*) or spec- *L. rhamnosus*, *M. orientalis,*

Specific requirements for growth fic nitrogen sources (e.g. *L. lactis* lacks genes for *de novo* *Pleurotus sajor-caju*^h^, *Saccharo-*

synthesis of some proteins), physicochemical conditions (see *mycopsis* *fodiens*, *S.cerevisiae,* *Extremophiles* below), or symbiotic, parasitic or microbial *S. ruber*, *Termitomyces* spp.

consortium interactions. Typically evade or minimize competition

by inhabiting hostile environments and/or utilizing resources that

are unused by the majority of the microbial community (e.g.

chemolithotrophs)

*Extremophiles* Optimal growth-rate occurs under Adapted to extreme conditions where competition for resources *Aspergillus wentii*, *Dunaliella*

one or more extreme conditions may be reduced as a consequence of limited microbial diversity *salina*, *Haloquadratum walsbyi*,

(e.g. low water activity, high  *Pyrolobus fumarii*, *Thiobacillus*

pressure, extreme temperature *thiooxidans*, *Xeromyces*

and/or pH) *bisporus*

*Mesophiles*^i^ Growth-rate optimal under non- Thrive in conditions that can support the majority of microbial *Caulobacter crescentus*^k^,

extreme conditions^j^, e.g. mid-range species *Metarhizium anisopliae*,

temperatures (~ 23–40°C) or high *Mycoplasma pneumoniae*,

water activity (~ 0.95–1) *Yarrowia lipolytica*

___________________________________________________________________________________________________________________________________________________________________________

**a.** These categories are not necessarily mutually exclusive.

**b.** Whereas species such as *Escherichia coli* and *Mycobacterium smegmatis* have some weed traits (see Tables 3, 5, and 6), they have a limited ability to dominate open habitats (Table S1). The expression ‘bacterial weeds’ has occasionally been used, from an anthropocentric viewpoint, to describe unwanted contaminants of laboratory cultures (such contaminants are typically copiotrophs, some of which have a low competitive ability).

**c.** Many non-weed species may be difficult to cultivate and, as a result, may be relatively little studied.

**d.** For example *Lactobacillus acidophilus* and *Lactococcus lactis* (Altermann *et al*., 2005; Dressaire *et al*., 2008).

**e.** Most frequently applied to bacterial species. See also footnote b.

**f.** See Fierer *et al*. (2007).

**g.** See (Gostinčar *et al*., 2011; Zalar *et al*., 2011).

**h.** *Pleurotus sajor-caju*, like many basidiomycetes, is a specialist in terms of nutritional as well as physicochemical requirements (Kashangura *et al*., 2006).

**i.** Microbes that grow only/optimally under non-extreme conditions such as those that favour mid-range temperature and xero-intolerant microbes (Gostinčar *et al*., 2010).

**j.** Environments that are extreme are defined according to the low proportion of microorganisms able to tolerant the associated conditions.

**k.** *Caulobacter crescentus* is unable to grow below a water activity of ~ 1 (Brown, 1990).

**Supporting References**

Altermann, E., Russell, W.M., Azcarate-Peril, M.A., Barrangou, R., Buck, B.L., McAuliffe, O., *et al*. (2005) Complete genome sequence of the probiotic lactic acid bacterium *Lactobacillus acidophilus* NCFM. *Proc Natl Acad Sci USA* **102:** 3906-3912.

Andrews, J.H., and Harris, R.F. (1986) R- and K-selection and microbial ecology. *Adv Microb Ecol* **9:** 99-148.

Antón, J., Oren, A., Benlloch, S., Rodríguez-Valera, F., Amann, R., and Rosselló-Mora, R. (2002) *Salinibacter ruber* gen. nov., sp. nov., a novel, extremely halophilic member of the *Bacteria* from saltern crystallizer ponds. *Int J Syst Evol Microbiol* **52:** 485-491.

Aquilanti, L., Santarelli, S., Silvestri, G., Osimani, A., Petruzzelli, A., and Clementi, F. (2007) The microbial ecology of a typical Italian salami during its natural fermentation. *Int J Food Microbiol* **120:** 136-145.

Atlas, R.M., and Bartha, R. (1993) *Microbial Ecology: Fundamentals and Applications*. 3rd edn. Redwood City, CA: The Benjamin/Cummings Publishing Company, Inc.

Baati, H., Guermazi, S., Amdouni, R., Gharsallah, N., Sghir, A., and Ammar, E. (2008) Prokaryotic diversity of a Tunisian multipond solar saltern. *Extremophiles* **12:** 505-518.

Bardavid, R.E., and Oren, A. (2008) Sensitivity of *Haloquadratum* and *Salinibacter* to antibiotics and other inhibitors: implications for the assessment of the contribution of Archaea and Bacteria to heterotrophic activities in hypersaline environments. *FEMS Microbiol Ecol* **63:** 309-315.

ben Omar, N., and Ampe, F. (2000) Microbial community dynamics during the production of the Mexican fermented maize dough Pozol. *Appl Environ Microbiol* **66:** 3664-3673.

Brown, A.D. (1990) *Microbial Water Stress Physiology – Principles and Perspectives.* Chichester, UK: Wiley.

Casalta, E., Sorba, J.M., Aigle, M., and Ogier, J.C. (2009) Diversity and dynamics of the microbial community during the manufacture of Calenzana, an artisanal Corsican cheese. *Int J Food Microbiol* **133:** 243-251.

Collado-Fabbri, S., Vaulot, D., and Ulloa, O. (2011) Structure and seasonal dynamics of the eukaryotic picophytoplankton community in a wind-driven coastal upwelling ecosystem. *Limnol and Oceanogr* **56:** 2334-2346.

Coutinho, T.A., and Venter, S.N. (2009) *Pantoea ananatis*: an unconventional plant pathogen. *Mol Plant Pathol* **10:** 325-335.

Daffonchio, D., Borin, S., Brusa, T., Brusetti, L., van der Wielen, P.W.J.J., Bolhuis, H., *et al*. (2006). Stratified prokaryote network in the oxic-anoxic transition of a deep sea halocline. *Nature* **440:** 203-207.

De Baere T., Verheslst R., Labit C., Verschraegen G., Wauters G., Clayes G., *et al*. (2004) Bacteremic infection with *Pantoea ananatis*. *J Clin Microbiol* **42:** 4393-4395.

de Pina, C.G., and Hogg, T.A. (1999) Microbial and chemical changes during the spontaneous ensilage of grape pomace. *J Appl Microbiol* **86:** 777-784.

Dressaire, C., Redon, E., Milhem, H., Besse, P., Loubière, P., and Cocaign-Bousquet, M. (2008) Growth rate regulated genes and their wide involvement in the *Lactococcus lactis* stress responses. *BMC Genomics* **9:** 343.

Fahlgren, C., Hagström, A., Nilsson, D., and Zweifel, U.L. (2010) Annual variations in the diversity, viability, and origin of airborne bacteria. *Appl Environ Microbiol* **76:** 3015-3025.

Ferrando, L., Fernández Mañay, J., Fernández Scavino, A. (2012) Molecular and culture-dependent analyses revealed similarities in the endophytic bacterial community composition of leaves from three rice (*Oryza sativa*) varieties. *FEMS Microbiol Ecol* **80:** 696-708.

Fierer, N., Bradford, M.A., and Jackson, R.B. (2007) Toward an ecological classification of soil bacteria. *Ecology* **88:** 1354-1364.

Ghai, R., Pašić, L., Fernández, A.B., Martin-Cuadrado, A.B., Mizuno, C.M., McMahon, K.D., *et al*. (2011) New abundant microbial groups in aquatic hypersaline environments. *Sci Rep* **1:** 135.

Gostinčar, C., Grube, M., and Gunde-Cimerman, N. (2011) Evolution of fungal pathogens in domestic environments? *Fungal Biol* **115:** 1008-1018.

Gostinčar, C., Grube, M., de Hoog, S., Zalar, P., and Gunde-Cimerman, N. (2010) Extremotolerance in fungi: evolution on the edge. *FEMS Microbiol Ecol* **71:** 2-11.

Greene, E.A., Kay, J.G., Jaber, K., Stehmeier, L.G., and Voordouw, G. (2000) Composition of soil microbial communities enriched on a mixture of aromatic hydrocarbons. *Appl Environ Microbiol* **66:** 5282-5289.

Haecky, P., Jonsson, S., and Andersson, A. (1998) Influence of sea ice on the composition of the spring phytoplankton bloom in the northern Baltic Sea. *Polar Biol* **20:** 1-8.

Hallsworth, J.E. (1998) Ethanol-induced water stress in yeast. *J Ferment Bioeng* **85:** 125-137.

Hameed, A.A.A., and Khodr, M.I. (2001) Suspended particulates and bioaerosols emitted from an agricultural non-point source. *J Environ Monit* **3:** 206-209.

Hunter, P.J., Hand, P., Pink, D., Whipps, J.M., and Bending, G.D. (2010) Both leaf properties and microbe-microbe interactions influence within-species variation in bacterial population diversity and structure in the lettuce (*Lactuca* species) phyllosphere. *Appl Environ Microbiol* **76:** 8117-8125.

Irum, F., Suhail, M., and Abro, H. (2007) Keratinophilic fungi from the soil of District, Jamshoro, Sindh, Pakistan. *Pak J Bot* **39:** 1377-1382.

Jones, E.B.G. (2011) Fifty years of marine mycology. *Fungal Divers* **50:** 73-112.

Kachalkin, A.V., and Yurkov, A.M. (2012) Yeast communities in Sphagnum phyllosphere along the temperature-moisture ecocline in the boreal forest-swamp ecosystem and description of *Candida sphagnicola* sp. nov.. *Antonie van Leeuwenhoek* **102:** 29-43.

Kaga, H., Mano, H., Tanaka, F., Watanabe, A., Kaneko, S., and Morisaki, H. (2009) Rice seeds as sources of endophytic bacteria. *Microbes Environ* **24:** 154-162.

Kashangura, C., Hallsworth, J.E., and Mswaka, A.Y. (2006). Phenotypic diversity amongst strains of *Pleurotus sajor-caju*: implications for cultivation in arid environments. *Mycol Res* **110:** 312-317.

Kazda, J., and Falkinham III, J.O. (2009) Mycobacteria in sphagnum, peats and potting soils. In *The Ecology of Mycobacteria: Impact on Animal’s and Human’s Health*. Kazda, J., Pavlik, I., Falkinham III, J.O., and Hruska K. (eds.) Heidelberg: Springer

Khemakhem, H., Elloumi, J., Moussa, M., Aleya, L., & Ayadi, H. (2010) The concept of ecological succession applied to phytoplankton over four consecutive years in five ponds featuring a salinity gradient. *Estuar Coast Shelf Sci* **88:** 33-44.

Koch, A.L. (2001) Oligotrophs versus copiotrophs. *Bioessays* **23:** 657-661.

Leão, P.N., Ramos, V., Vale, M., Machado, J.P., and Vasconcelos, V.M. (2012) Microbial community changes elicited by exposure to cyanobacterial allelochemicals. *Microb Ecol* **63:** 85-95.

Louvel, B., Cébron, A., and Leyval, C. (2011) Root exudates affect phenanthrene biodegradation, bacterial community and functional gene expression in sand microcosms. *Int Biodeter Biodegr* **65:** 947-953.

Margesin, R., Labbe, D., Schinner, F., Greer, C.W., and Whyte, L.G. (2003) Characterization of hydrocarbon-degrading microbial populations in contaminated and pristine alpine soils. *Appl Environ Microbiol* **69:** 3085-3092.

Mazzola, M. (1999) Transformation of soil microbial community structure and *Rhizoctonia*-suppressive potential in response to apple roots. *Phytopathology* **89:** 920-927.

Michel, C., Nielsen, T.G., Nozais, C., and Gosselin, M. (2002) Significance of sedimentation and grazing by ice micro- and meiofauna for carbon cycling in annual sea ice (northern Baffin Bay). *Aquat Microb Ecol* **30:** 57-68.

Morrison-Gardiner, S. (2002) Dominant fungi from Australian coral reefs. *Fungal Divers* **9:** 105-121.

Morrissey, W.F., Davenport, B., Querol, A., and Dobson, A.D.W. (2004) The role of indigenous yeasts in traditional Irish cider fermentations. *J Appl Microbiol* **97:** 647-655.

Nguyen-Viet, H., Gilbert, D., Mitchell, E.A.D., Badot, P.M., and Bernard, N. (2007) Effects of experimental lead pollution on the microbial communities associated with *Sphagnum fallax* (Bryophyta). *Microb Ecol* **54:** 232-241.

Nisiotou, A.A., Chorianopoulos, N., Nychas, G.-J.E., and Panagou, E.Z. (2010) Yeast heterogeneity during spontaneous fermentation of black Conservolea olives in different brine solutions. *J Appl Microbiol* **108:** 396-405.

Nunes, F.M.N., Veloso, M.C.C., Pereira, P.A.P., and de Andrade, J.B. (2005) Gas-phase ozonolysis of the monoterpenoids (*S*)-(+)-carvone, (*R*)-(-)-carvone, (-)-carveol, geraniol and citral. *Atmos Environ* **39:** 7715-7730.

Ogino, A., Koshikawa, H., Nakahara, T., and Uchiyama, H. (2001) Succession of microbial communities during a biostimulation process as evaluated by DGGE and clone library analyses. *J Appl Microbiol* **91**: 625-635.

Oh, D., Porter, K., Russ, B., Burns, D., Dyall-Smith, M. (2010) Diversity of *Haloquadratum* and other haloarchaea in three, geographically distant, Australian saltern crystallizer ponds. *Extremophiles* **14:** 161-169.

Olstorpe, M., Borling, J., Schnürer, J., and Passoth, V. (2010) *Pichia anomala* yeast improves feed hygiene during storage of moist crimped barley grain under Swedish farm conditions. *Anim Feed Sci Technol* **156:** 47-56.

Peu, P., Brugère, H., Pourcher, A.-M., Kérourédan, M., Godon, J.-J., Delgenès, J.-P., *et al*. (2006) Dynamics of a pig slurry microbial community during anaerobic storage and management. *Appl Environ Microbiol* **72:** 3578-3585.

Pretorius, I.S. (2000) Tailoring wine yeast for the new millennium: novel approaches to the ancient art of winemaking. *Yeast* **16:** 675-729.

Ramos, C.L., de Almeida, E.G., de Melo Pereira, G.V., Cardoso, P.G., Dias, E.S., and Schwan, R.F. (2010) Determination of dynamic characteristics of microbiota in a fermented beverage produced by Brazilian Amerindians using culture-dependent and culture-independent methods. *Int J Food Microbiol* **140:** 225-231.

Randazzo, C.L., Pitino, I., Ribbera, A., and Caggia C. (2010) Pecorino Crotonese cheese: Study of bacterial population and flavour compounds. *Food Microbiol* **27:** 363-374.

Rao, D., Webb, J.S., and Kjelleberg, S. (2005) Competitive interactions in mixed-species biofilms containing the marine bacterium *Pseudoalteromonas tunicate*. *Appl Environ Microbiol* **71:** 1729-1736.

Röling, W.F.M., Milner, M.G., Jones, D.M., Fratepietro, F., Swannell, R.P.J., and Head, I.M. (2004) Bacterial community dynamics and hydrocarbon degradation during a field-scale evaluation of bioremediation on a mudflat beach contaminated with buried oil. *Appl Environ Microbiol* **70:** 2603-2613.

Schloss, P.D., Hay, A.G., Wilson, D.B., and Walker, L.P. (2003) Tracking temporal changes of bacterial community fingerprints during the initial stages of composting. *FEMS Microbiol Ecol* **46:** 1-9.

Senthilkumar, K., Udaiyan, K., and Manian, S. (1993) Successional pattern of mycoflora associated with litter degradation in a *Cymbopogon caesius*-dominated tropical grassland. *Trop Grasslands* **27:** 121-127.

Smith, M.L., Bruhn, J.N., Anderson, J.B. (1992) The fungus *Armillaria bulbosa* is among the largest and oldest living organisms. *Nature* **356:** 428-431.

Thomas, R., Grimsley, N., Escande, M.L., Subirana, L., Derelle, E., and Moreau, H. (2011) Acquisition and maintenance of resistance to viruses in eukaryotic phytoplankton populations. *Environ Microbiol* **13:** 1412-1420.

Trigal, C., Goedkoop, W., and Johnson, R.K. (2011) Changes in phytoplankton, benthic invertebrate and fish assemblages of boreal lakes following invasion by *Gonyostomum semen*. *Freshw Biol* **56:** 1937-1948.

Wheeler, K.A., and Hocking, A.D. (1993) Interactions among xerophilic fungi associated with dried salted fish. *J Appl Bacteriol* **74:** 164-169.

Wittmann, C., Zeng, A.P., and Deckwer, W.D. (1998) Physiological characterization and cultivation strategies of the pentachlorophenol-degrading bacteria *Sphingomonas chlorophenolica* RA2 and *Mycobacterium chlorophenolicum* PCP-1. *J Ind Microbiol Biotechnol* **21:** 315-321.

Womack, A.M., Bohannan, B.J.M., and Green, J.L. (2010) Biodiversity and biogeography of the atmosphere. *Phil Trans R Soc B* **365:** 3645-3653.

Womack, A.M., Artaxo, P.E., Ishida, F., Jardine, K.J., Saleska, S.R., Wiedemann, K.T., *et al.* (2012) Microbial community composition and gene expression in the atmosphere over the Brazilian Amazon. *ASM, 112th General Meeting American Society for Microbiology, San Francisco, California.*

Zalar, P., Novak, M., De Hoog, G.S., and Gunde-Cimerman, N. (2011) Dishwashers - A man-made ecological niche accommodating human opportunistic fungal pathogens. *Fungal Biol* **115:** 997-1007.

Zhou, Z.-X., Jiang, H., Yang, C., Yang, M.-Z., and Zhang, H.-B. (2010) Microbial community on healthy and diseased leaves of an invasive plant *Eupatorium adenophorum* in Southwest China. *J Microbiol* **48:** 139-145.
